# Branching out at *C*-2 of septanosides. Synthesis of 2-deoxy-2-*C*-alkyl/aryl septanosides from a bromo-oxepine

**DOI:** 10.3762/bjoc.8.59

**Published:** 2012-04-10

**Authors:** Supriya Dey, Narayanaswamy Jayaraman

**Affiliations:** 1 Department of Organic Chemistry, Indian Institute of Science, Bangalore 560 012, India

**Keywords:** C–C bond formations, 2-deoxy sugars, organometallic reactions, septanosides, unsaturated sugars

## Abstract

This paper deals with the synthesis of 2-deoxy-2-*C*-alkyl/aryl septanosides. A range of such septanoside derivatives was synthesized by using a common bromo-oxepine intermediate, involving C–C bond forming organometallic reactions. Unsaturated, seven-membered septanoside vinyl bromides or bromo-oxepines, obtained through a ring expansion methodology of the cyclopropane derivatives of oxyglycals, displayed a good reactivity towards several acceptor moieties in C–C bond forming Heck, Suzuki and Sonogashira coupling reactions, thus affording 2-deoxy-2-*C*-alkyl/aryl septanosides. Whereas Heck and Sonogashira coupling reactions afforded 2-deoxy-2-*C*-alkenyl and -alkynyl derivatives, respectively, the Suzuki reaction afforded 2-deoxy-2-*C*-aryl septanosides. Deprotection and reduction of the 2-deoxy-2-alkenyl derivative afforded the corresponding 2-deoxy-2-*C*-alkyl septanoside free of protecting groups. The present study illustrates the reactivity of bromo-oxepine in the synthesis of hitherto unknown septanosides, branching out at *C*-2, through C–C bond formation with alkyl and aryl substituents.

## Introduction

Septanoses and septanosides are unnatural, seven-membered cyclic sugars [1]. Methods of preparation and the exploration of the properties of these unnatural sugars are of high interest [2]. An early isolation of septanose was achieved through cyclization of generic hexose sugars, which afforded minor amounts of septanose, along with furanose and pyranose, which formed in major amounts [3]. Synthetic approaches to septanoses have been explored in many instances, for example, (i) hemiacetal or acetal formation from a linear precursor containing aldehyde and an appropriately positioned hydroxyl group [4–8]; (ii) Knoevenagel-type condensation of sugar aldehyde with active methylene compounds [9–10]; (iii) ring-closing metathesis reactions of appropriately installed diene derivatives [11–13]; (iv) ring expansion of 1,2-cyclopropanated sugars [14–17]; (v) Baeyer–Villiger oxidation of inositol derivatives [18–19] and (vi) electrophile-induced cyclization [20]. We recently developed a new methodology to prepare septanosides, which involved a sequence of dihalocarbene insertion on to an oxyglycal, ring opening of the cyclopropyl moiety with a nucleophile, and oxidation and reduction reactions, so as to permit the expansion of six-membered pyranoses to seven-membered septanosides [21–23]. Features of this methodology include the formation of vinyl halide, vinyl ether, diketone and diol intermediates, which are potential sites for varied types of functionalizations. While exploring such features, we undertook the preparation of septanoside derivatives that are branched out at *C*-2, so as to afford 2-deoxy-2-*C*-alkyl/aryl derivatives, through C–C bond formations mediated by organometallic reagents. Details of the preparation of 2-deoxy-2-*C*-aryl/alkyl septanosides are described herein.

## Results and Discussion

**Figure 1:**
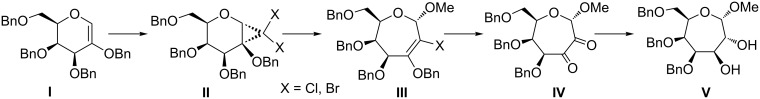
Synthetic route to transform oxyglycal **I** to a septanoside **V**.

The methodology of septanoside preparation starting from an oxyglycal is shown in Figure 1 [21]. The oxygen at *C*-2 of oxyglycal **I** was retained throughout until the septanoside **V** was obtained. More importantly, vinyl halide **III** and diketone **IV** also form as intermediates of the reaction and these intermediates provide an avenue to expand the scope of the reaction sequence.

**Scheme 1:**
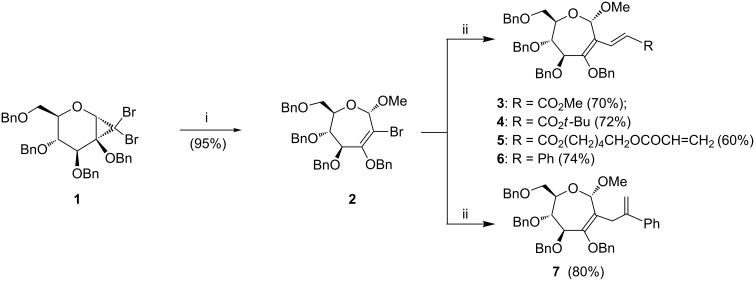
Reaction conditions: (i) NaOMe, PhMe, reflux, 8 h; (ii) methyl acrylate (for **3**); *tert*-butyl acrylate (for **4**); H_2_C=CHCOO(CH_2_)_5_OCOCH=CH_2_ (for **5**; **2**/substrate = 1:0.57); styrene (for **6**); α-methyl styrene (for **7**); Pd(OAc)_2_ (10 mol %), Cs_2_CO_3_ (1.5 molar equiv), 1,4-dioxane, 98 °C, 72 h.

In the present work, we envisaged that **III** would form as a synthon to implement C–C bond forming reactions. Vinyl halide **2** was synthesized through a ring-expansion reaction of cyclopropanated adduct **1** (Scheme 1), as reported previously [21]. The reactivity at *C*-2 of **2** was examined by the chosen organometallic reactions, namely, Heck, Suzuki and Sonogashira coupling reactions. Heck coupling reactions [24–25] were undertaken first. Thus, the reaction of bromo-oxepine **2** with methyl acrylate was performed, in the presence of Pd(OAc)_2_ (10 mol %) and Cs_2_CO_3_ in 1,4-dioxane, at 98 °C (Scheme 1), to afford diene **3**, in 70% yield. The presence of doublets at 7.80 and 5.97 ppm (*J* = 16.0 Hz) in the ^1^H NMR spectrum and signals at 136.3 ppm and 119.5 ppm in the ^13^C NMR spectrum confirmed the formation of **3**.

Having realized the synthesis of one product, reactions of **2** were performed with a few other substrates, namely, *tert*-butyl acrylate, a substrate presenting two acrylates within the molecule, styrene, and α-methyl styrene (Scheme 1). Reactions with these substrates also afforded the diene products **4**–**7**, in good yields. The anticipated two Heck coupling reactions with the substrate that presents two acrylates, could not be achieved, rather only the mono-Heck coupling product **5** was obtained. Alternative reaction conditions were attempted, for example, by using Pd(PPh_3_)_2_Cl_2_ (10 mol %), instead of Pd(OAc)_2_, while keeping other parameters of the reaction uniform, yet the double-Heck coupling product was not observed. The newly generated exocyclic olefin protons resonated as two distinct doublets in **4** at 7.72 and 5.88 ppm (*J* = 16.4 Hz); in **5** at 7.79 and 5.93 ppm (*J* = 16.4 Hz) and in **6** at 7.19 and 6.66 ppm (*J* = 16.8 Hz). Further, the exocyclic double-bond carbon nuclei resonated at ~136–130 and ~122 ppm in the ^13^C NMR spectra of **4**–**6**. The reactions afforded only the (*E*)-isomer. Interestingly, when the reaction was performed with α-methyl styrene, product **7**, with an exocyclic double bond isomerization to a terminal double bond was observed. The appearance of two singlets at 5.30 and 5.14 ppm in the ^1^H NMR spectrum indicated the presence of two vinylic protons in **7**. On the other hand, the exocyclic methylene moiety at *C*-2 in **7** appeared as two distinct doublets (3.88, 3.08 ppm, *J* = 14.4 Hz) in the ^1^H NMR spectrum. Further structural assignments of **7** were performed through COSY and HSQC experiments.

**Scheme 2:**
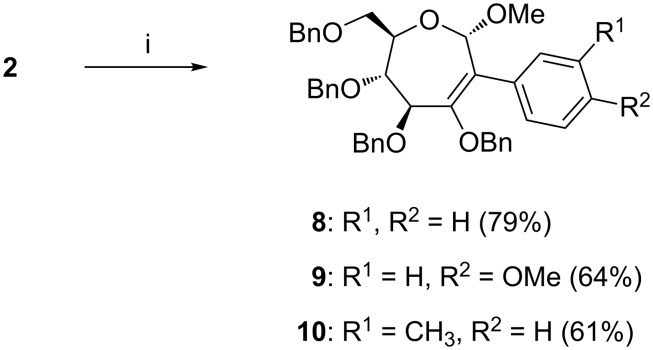
Reaction conditions: (i) phenylboronic acid (for **8**); 4-methoxyphenylboronic acid (for **9**); 3-methylphenylboronic acid (for **10**); Pd(OAc)_2_ (10 mol %), Cs_2_CO_3_ (1.5 molar equiv), 1,4-dioxane, 98 °C, 72 h.

Following the Heck coupling reactions on bromo-oxepine **2**, efforts were undertaken to implement C–C bond forming Suzuki and Sonogashira coupling reactions. Suzuki reactions were undertaken by involving phenylboronic acid and substituted phenylboronic acids [26–27], in the presence of Pd(OAc)_2_ (10 mol %) and Cs_2_CO_3_ in 1,4-dioxane at 98 °C (Scheme 2). The reactions afforded septanosides **8–10**, which are derivatized with a phenyl substituent at *C*-2, in moderate yields. The formation of a C–C bond at *C*-2 in **8–10** was inferred by the observation of shifts of the *C*-2 nuclei signal at ~129 ppm, which in the case of bromo-oxepine was observed at 114.3 ppm. Analyses of ^1^H and ^13^C NMR spectra and mass spectra confirmed the constitution of **8–10**.

**Scheme 3:**
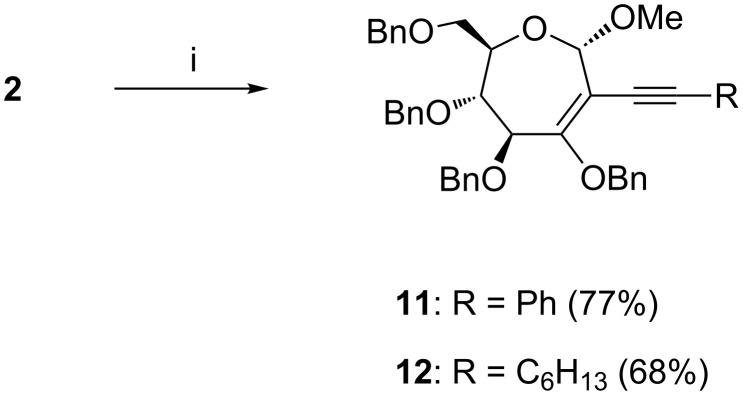
Reaction conditions: (i) phenylacetylene (for **11**); oct-1-yne (for **12**); Pd(PPh_3_)_2_Cl_2_ (20 mol %), CuI (10 mol %), DMF/THF/Et_3_N 3:2:1, 98 °C, 72 h.

The reactivity of bromo-oxepine, the key intermediate of the septanoside synthesis earlier, was explored further in the context of C–C bond formation at *C*-2, through another versatile C–C bond forming reaction, namely, Sonogashira coupling [28–29]. Reactions of **2** with acetylenes were performed in the presence of Pd(PPh_3_)_2_Cl_2_ (20 mol %) and CuI (10 mol %) in a DMF/THF/Et_3_N 5:3:2 solvent mixture as the optimized protocol. The use of Pd(OAc)_2_ as a catalyst or Et_3_N as the base did not promote the reaction, leading only to the recovery of the starting material. Thus, the reaction of **2** with phenylacetylene and oct-1-yne led to the formation of the corresponding 2-deoxy-2-*C*-alkynyl septanosides **11** and **12** (Scheme 3) in moderate yields. Prolonging the reaction time and increasing the catalyst loading did not increase the yields, although dehalogenation of **2** to oxepine was found to occur to a minor extent when the reaction time was increased to several days. ^13^C NMR spectra of **11** and **12** showed resonances for the newly formed C–C bond at **11**: 108.4 ppm (*C*-2) and 95.8 ppm (*C*≡C-Ph); **12**: 109.9 ppm (*C*-2) and 97.4 ppm (*C*≡C-C_6_H_13_). Further, ^1^H and ^13^C NMR spectroscopic and mass spectrometric analyses confirmed the constitutions of **11** and **12**.

**Scheme 4:**
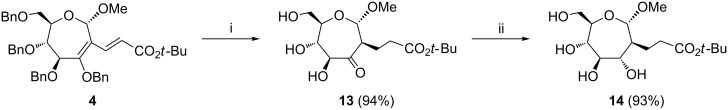
Reaction conditions: (i) Pd/C (10 %), H_2_, MeOH, rt, 24 h; (ii) NaBH_4_, MeOH, 0 °C to rt, 3 h.

Having observed a good reactivity of bromo-oxepine **2** in C–C bond forming reactions, we used one of the 2-deoxy-2-*C*-alkyl derivatives, namely, product **4** for further reactions, leading to a 2-deoxy-2-*C*-alkyl septanoside containing free hydroxyl groups. Towards this effort, **4** was subjected first to a hydrogenolysis (Pd/C, H_2_), which afforded D**-***manno***-**sept-3-uloside **13** as single diastereomer in good yield (Scheme 4).

The configuration of C-2 in **13** was confirmed through HMQC and COSY experiments. A doublet at 4.37 ppm with *J*_H1,H2_ of 8.0 Hz indicated a *trans*-configuration of H-2 with respect to H-1 in **13**. The presence of sets of protons in the ^1^H NMR spectrum, one at 2.05 and 1.78 ppm (multiplet) and the other at 2.21 ppm (t, *J* = 7.4 Hz), corresponding to exocyclic methylene moieties in **13**, resulting from the concomitant reduction of the exocyclic double bond in **4**, was also observed. The presence of the ketone functionality in **13** was inferred from the resonance at 208.4 ppm in the ^13^C NMR spectrum. Subsequent to hydrogenolysis, the treatment of **13** with NaBH_4_ facilitated the reduction of the keto-moiety to the corresponding alcohol **14**, in an excellent yield. The *trans*-bisequatorial configuration of the hydroxyl groups at *C*-3 and *C*-4 in **14** was inferred from a ^3^*J*_H3,H4_ of 12.4 Hz, in the ^1^H NMR spectrum. On the other hand, the proton at *C*-2 merged with the exocyclic methylene group, leading to an inability to assess the H-2,H-3 coupling constant in **14**. Having defined the configuration of the substituent at *C*-2 in **13**, we infer a *trans*-configuration of the substituent at *C*-2 and *C*-3. The results of mass spectrometric analysis concurred with the constitutions of **13** and **14**.

## Conclusion

The present study illustrates the effective application of synthetically useful bromo-oxepine for the preparation of hitherto unknown 2-deoxy-2-*C*-alkyl/aryl septanoside derivatives. C–C bond forming Heck, Suzuki and Sonogashira coupling reactions, with appropriate acrylates, arylboronic acids and alkynes, afforded the respective cross-coupled products in good yields. It is pertinent to note that the implementation of such reactions is known in seven-membered 1,2-oxazepines, so as to secure the corresponding cross-coupling products [30]. Furthermore, one of the 2-deoxy-2-*C*-alkyl septanoside derivatives was converted to a hydroxyl-group-free methyl 2-deoxy-2-*C*-alkyl septanoside. The present study illustrates the scope of seven-membered bromo-oxepines as useful substrates for the generation of 2-deoxy-2-*C*-alkyl/aryl septanosides, in addition to our previous efforts to progress such intermediates to a number septanosides and septanoside-containing di- and tri-saccharides.

## Experimental

### General

Chemicals were purchased from commercial sources and were used without further purification. Solvents were dried and distilled according literature procedures. Analytical TLC was performed on commercial Merck plates coated with silica gel GF254 (0.25 mm). Silica gel (230–400 mesh) was used for column chromatography. Optical rotations were recorded on a JASCO Model P-1020 polarimeter at the sodium D line at 24 °C. High-resolution mass spectra were obtained from a Q-TOF instrument by the electrospray ionization (ESI) technique. ^1^H and ^13^C NMR spectral analyses were performed on 400 MHz and 100 MHz spectrometers, respectively, with the residual solvent signal acting as the internal standard. COSY and HSQC analyses were performed on a 400 MHz NMR spectrometer.

**Methyl 2-deoxy-2-*C*-(2-(*tert*-butoxycarbonyl)vinyl)-3,4,5,7-*tetra*-*O*-benzyl-α-D-*arabino*-hept-2-enoseptanoside (4):** A solution of **2** [21] (0.05 g, 0.07 mmol) in 1,4-dioxane (1 mL) was admixed with Pd(OAc)_2_ (1 mg, 10 mol %) under a N_2_ atmosphere, and this was followed by the addition of Cs_2_CO_3_ (0.03 g, 0.11 mmol) and *tert*-butyl acrylate (0.02 mL, 0.153 mmol), in a sealed tube. The reaction mixture was stirred at 98 °C for 72 h, cooled, filtered, diluted with EtOAc (20 mL), washed with water (2 × 30 mL) and brine (2 × 10 mL), dried (Na_2_SO_4_) and concentrated in vacuo. The resulting residue was purified (hexane/EtOAc 9:1) to afford **4** (0.038 g, 72%), as an oil. *R*_f_ 0.48 (hexane/EtOAc 9:1); [α]_D_ −130.8 (*c* 1.0, CHCl_3_); ^1^H NMR (400 MHz, CDCl_3_) δ 7.72 (d, *J* = 16.4 Hz, 1H, -C*H*=CHCO_2_*t*-Bu), 7.33–7.24 (m, 18H, aromatic), 7.10–7.08 (m, 2H, aromatic), 5.88 (d, *J* = 16.4 Hz, 1H, -CH=C*H*CO_2_*t*-Bu), 5.36 (s, 1H, H-1), 4.69–4.56 (m, 4H, PhC*H*_2_), 4.48 (d, *J* = 12.0 Hz, 1H, PhC*H*_2_), 4.43 (d, *J* = 12.0 Hz, 1H, PhC*H*_2_), 4.33 (d, *J* = 12.0 Hz, 1H, PhC*H*_2_), 4.24 (d, *J* = 12.0 Hz, 1H, PhC*H*_2_), 4.21–4.17 (m, 2H, H-4 and H-6), 3.75 (dd, *J* = 8.4, 1.4 Hz, 1H, H-5), 3.63–3.57 (br, 1H, H-7a), 3.53–3.52 (br, 1H, H-7b), 3.51 (s, 3H, OMe), 1.47 (s, 9H, *t*-Bu); ^13^C NMR (100 MHz, CDCl_3_) δ 166.6 (C=O), 158.7 (C-3), 138.2–137.0 (aromatic), 136.4 (-*C*H=CHCO_2_*t*-Bu), 128.4–127.5 (aromatic ), 124.4 (C-2), 121.9 (-CH=*C*HCO_2_*t*-Bu), 100.0 (C-1), 80.1 (C-5), 79.9 (C-4), 73.0 (Ph*C*H_2_), 72.8 (Ph*C*H_2_), 72.0 (Ph*C*H_2_), 71.2 (Ph*C*H_2_), 71.0 (C-6), 70.8 (C-7), 55.5 (OMe), 28.1 (*t*-Bu); HRMS–ESI (*m*/*z*): [M + Na]^+^ calcd for 715.3247; found, 715.3245.

**Methyl 2-deoxy-2-*C*-(2-phenylallyl)-3,4,5,7-tetra-*O-*benzyl-α-D-*arabino*-hept-2-enoseptanoside (7):** A solution of **2** [21] (0.05 g, 0.07 mmol) in 1,4-dioxane (1 mL) was admixed with Pd(OAc)_2_ (1 mg, 10 mol %) under a N_2_ atmosphere, and was followed by the addition of Cs_2_CO_3_ (0.03 g, 0.11 mmol) and α-methyl styrene (0.01 mL, 0.09 mmol) in a sealed tube. The reaction mixture was stirred at 98 °C for 72 h, cooled, filtered, diluted with EtOAc (20 mL), washed with water (2 × 30 mL) and brine (2 × 10 mL), dried (Na_2_SO_4_) and concentrated in vacuo. The crude reaction mixture was purified (hexane/EtOAc 92:8) to afford **7** (0.042 g, 80%), as an oil. *R*_f_ 0.60 (hexane/EtOAc 9:1); [α]_D_ −58.8 (*c* 0.5, CHCl_3_); ^1^H NMR (400 MHz, CDCl_3_) δ 7.44 (d, *J* = 7.6 Hz, 2H, aromatic), 7.33–7.21 (m, 21H, aromatic), 7.18 (d, *J* = 4.8 Hz, 2H, aromatic), 5.30 (app. s, 1H, C*H*H=CPh), 5.14 (app. s, 1H, CH*H*=CPh), 4.98 (s, 1H, H-1), 4.58 (d, *J* = 12.4 Hz, 2H, PhC*H*_2_), 4.44 (d, *J* = 12.0 Hz, 2H, PhC*H*_2_), 4.31 (d, *J* = 10.8 Hz, 2H, PhC*H*_2_), 4.22 (d, *J* = 11.6 Hz, 1H, PhC*H*_2_), 4.08–4.03 (band, 3H, H-4, H-6 and PhC*H*_2_), 3.88 (d, *J* = 14.4 Hz, 1H, -C*H*H *C*(Ph)=CH_2_), 3.61 (dd, *J* = 9.2, 1.6 Hz, 1H, H-5), 3.55 (dd, *J* = 10.4, 6.4 Hz, 1H, H-7a), 3.49 (dd, *J* = 8.8, 2.0 Hz, 1H, H-7b), 3.36 (s, 3H, OMe), 3.08 (d, *J* = 14.4 Hz, 1H, -CH*H C*(Ph)=CH_2_); ^13^C NMR (100 MHz, CDCl_3_) δ 150.9 (C-3), 146.3 (CH_2_-*C*(Ph)=CH_2_), 141.0–137.3 (aromatic), 128.3–126.6 (aromatic), 126.5 (C-2), 114.1 (C-10), 101.0 (C-1), 80.8 (C-5), 76.3 (C-4), 72.9 (Ph*C*H_2_), 72.0 (Ph*C*H_2_), 71.7 (Ph*C*H_2_), 71.3 (Ph*C*H_2_), 71.2 (C-7), 70.0 (C-6), 55.7 (OMe) 33.2 (-*CH_2_*-C(Ph)=CH_2_); HRMS–ESI (*m*/*z*): [M + Na]^+^ calcd for 705.3192; found, 705.3193.

**Methyl 2-deoxy-2-*C*-(*p*-methoxyphenyl)-3,4,5,7-tetra-*O-*benzyl-α-D-*arabino*-hept-2-enoseptanoside (9):** A solution of **2** [21] (0.05 g, 0.07 mmol) in 1,4-dioxane (1 mL) was admixed with Pd(OAc)_2_ (1 mg, 10 mol %) under a N_2_ atmosphere, and was followed by the addition of Cs_2_CO_3_ (0.03 g, 0.11 mmol) and 4-methoxyphenylboronic acid (0.012 g, 0.07 mmol), in a sealed tube. The reaction mixture was stirred at 98 °C for 72 h, cooled, filtered, diluted with EtOAc (20 mL), washed with water (2 × 30 mL) and brine (2 × 10 mL), dried (Na_2_SO_4_) and concentrated in vacuo. The crude reaction mixture was purified (hexane/EtOAc 8:2) to afford **9** (0.033 g, 64%), as an oil. *R*_f_ 0.60 (hexane/EtOAc 8:2); [α]_D_ −9.8 (*c* 0.1, CHCl_3_); ^1^H NMR (400 MHz, CDCl_3_) δ 7.38–7.11 (m, 20H, aromatic), 6.88 (d, *J* = 7.6 Hz, 2H, aromatic), 6.83 (d, *J* = 8.8 Hz, 2H, aromatic), 5.37 (s, 1H, H-1), 4.81 (d, *J* = 12.4 Hz, 1H, PhC*H*_2_), 4.64–4.49 (m, 3H, PhC*H*_2_), 4.40 (d, *J* = 11.6 Hz, 2H, PhC*H*_2_), 4.32–4.29 (br, 1H, H-6), 4.28 (app. d, *J* = 11.2 Hz, 1H, H-4), 4.23 (s, 2H, PhC*H*_2_), 3.80 (s, 3H, OMe), 3.77–3.74 (br, 1H, H-5), 3.66 (dd, *J* = 10.6, 6.4 Hz, 1H, H-7a), 3.58 (dd, *J* = 10.6, 2.4 Hz, 1H, H-7b), 3.33 (s, 3H, OMe); ^13^C NMR (100 MHz, CDCl_3_) δ 158.4 (aromatic), 152.3 (C-3), 138.4–137.3 (aromatic), 130.9 (aromatic), 129.0 (C-2), 128.4–127.4 (aromatic), 113.1 (aromatic), 102.2 (C-1), 80.7 (C-5), 78.3 (C-4), 73.0 (Ph*C*H_2_), 72.6 (Ph*C*H_2_), 72.0 (Ph*C*H_2_), 71.2 (C-6), 71.1 (C-7), 55.9 (OMe), 55.2 (-C_6_H_4_OMe); HRMS–ESI (*m*/*z*): [M + Na]^+^ calcd for 695.2985; found, 695.2983.

**Methyl 2-deoxy-2-*C*-(octyn-1-yl)-3,4,5,7-tetra-*O*-benzyl-α-D-*arabino*-hept-2-enoseptanoside (12):** A solution of **2** [21] (0.05 g, 0.07 mmol) in DMF/THF/Et_3_N 5:3:2 (1 mL) was admixed with Pd(PPh_3_)_2_Cl_2_ (0.01 g, 20 mol %) under a N_2_ atmosphere, and was followed by the addition of CuI (0.012 g, 10 mol %) and 1-octyne (0.023 mL, 0.14 mmol), in a sealed tube. The reaction mixture was stirred at 98 °C for 72 h, cooled, filtered, diluted with EtOAc (20 mL), washed with water (2 × 30 mL) and brine (2 × 10 mL), dried (Na_2_SO_4_) and concentrated in vacuo. The crude reaction mixture was purified (hexane/EtOAc 9:1) to afford **12** (0.035 g, 68%), as an oil. *R*_f_ 0.30 (hexane/EtOAc 9:1); [α]_D_ +4.56 (*c* 0.1, CHCl_3_); ^1^H NMR (400 MHz, CDCl_3_) δ 7.36–7.21 (m, 18H, aromatic), 7.07–7.05 (m, 2H, aromatic), 5.25 (s, 1H, H-1), 5.08 (d, *J* = 11.6 Hz, 1H, PhC*H*_2_), 4.80 (d, *J* = 11.6 Hz, 1H, PhC*H*_2_), 4.72 (d, *J* = 12.4 Hz, 1H, PhC*H*_2_), 4.60 (d, *J* = 12.4 Hz, 1H, PhC*H*_2_), 4.45 (d, *J* = 12.4 Hz, 2H, PhC*H*_2_), 4.32 (d, *J* = 11.6 Hz, 1H, PhC*H*_2_), 4.17 (d, *J* = 11.6 Hz, 1H, PhC*H*_2_), 4.14–4.10 (band, 2H, H-4 and H-6), 3.68 (dd, *J* = 8.8, 2.0 Hz, 1H, H-5), 3.60 (dd, *J* = 6.0, 3.0 Hz, 1H, H-7a), 3.52–3.49 (br, 1H, H-7b), 3.48 (s, 3H, OMe), 2.37 (t, *J* = 7.2 Hz, 2H, -C≡CC*H*_2_-), 1.55–1.51 (m, 1H, -C≡CCH_2_C*H*_2_-), 1.42–1.35 (m, 1H, -C≡CCH_2_C*H*_2_-), 1.30–1.18 (m, 6H, -C≡C(CH_2_)_2_(C*H*_2_)_3_-), 0.86 (t, *J* = 6.8 Hz, 3H, -C≡C(CH_2_)_5_*C*H_3_); ^13^C NMR (100 MHz, CDCl_3_) δ 160.9 (C-3), 138.4–137.5 (aromatic), 128.4–127.4 (aromatic), 109.9 (C-2), 100.8 (C-1), 97.4 (-*C*≡CCH_2_C*H*_2_-), 80.4 (C-5), 78.6 (C-4), 75.7 (-C≡*C*CH_2_CH_2_-), 73.3 (Ph*C*H_2_), 72.8 (Ph*C*H_2_), 71.8 (Ph*C*H_2_), 71.2 (Ph*C*H_2_), 71.1 (C-6), 70.8 (C-7), 55.9 (OMe), 31.3 (-C≡C(CH_2_)_3_*C*H_2_-), 28.6 (-C≡C(CH_2_)_4_*C*H_2_-), 28.5 (-C≡CCH_2_*C*H_2_-), 22.5 (-C≡C(CH_2_)_2_*C*H_2_-), 20.0 (-C≡C*C*H_2_(CH_2_)_4_CH_3_), 14.3 (-C≡C(CH_2_)_5_*C*H_3_); HRMS–ESI (*m*/*z*): [M + Na]^+^ calcd for 697.3505; found, 697.3507.

**Methyl 2-deoxy-2-*C-*(2-(*tert*-butoxycarbonyl)ethyl)-α-D-*manno*-sept*-*3-uloside (13):** A mixture of **4** (0.038 g, 0.054 mmol) and Pd/C (10%, 0.030 g) in MeOH (10 mL) was stirred under a positive pressure of H_2_ for 24 h at rt, filtered through a celite pad, and washed with MeOH (2 × 15 mL), and the solvents were removed in vacuo to afford **13** (0.017 g, 94%), as an oil. *R*_f_ 0.3 (MeOH/CHCl_3_ 1:1); [α]_D_ +63.12 (*c* 0.5, MeOH); ^1^H NMR (400 MHz, CD_3_OD) δ 4.37 (d, *J* = 8.0 Hz, 1H, H-1), 4.28 (app. d, *J* = 7.6 Hz, 1H, H-4), 4.08 (m, 1H, H-6), 3.85 (dd, *J* = 13.6, 2.4 Hz, 1H, H-7a), 3.73 (dd, *J* = 13.6, 4.8 Hz, 1H, H-7b), 3.46 (s, 3H, OMe), 3.35 (br, 1H, H-5), 3.22–3.17 (m, 1H, H-2), 2.21 (t, *J* = 7.4 Hz, 2H, -CH*_2_*-C*H*_2_CO_2_*t*-Bu), 2.10–2.01 (m, 1H, -C*H*HCH_2_CO_2_*t*-Bu), 1.83–1.75 (m, 1H, -CH*H*CH_2_CO_2_*t*-Bu), 1.49 (s, 9H, *t*-Bu); ^13^C NMR (100 MHz, CD_3_OD) δ 208.4 (C-3), 175.3 (C=O), 103.4 (C-1), 84.5 (C-4), 83.1 (C-*t*-Bu), 73.2 (C-5), 71.0 (C-6), 62.7 (C-7), 56.6 (OMe), 52.2 (C-2), 33.9 (-CH_2_*C*H_2_CO_2_*t*-Bu), 28.2 (*t*-Bu), 23.3 (-*C*H_2_CH_2_CO_2_*t*-Bu); HRMS–ESI (*m*/*z*): [M + Na]^+^ calcd for 357.1525; found, 357.1526.

## Supporting Information

File 1 Experimental procedures and spectroscopic data.
