# Supplementary material for: Branching out at C-2 of septanosides. Synthesis of 2-deoxy-2-C-alkyl/aryl septanosides from a bromo-oxepine
Source: Beilstein J Org Chem. 2012 Apr 10;8:522–7. doi: 10.3762/bjoc.8.59 (PMC3326632; doi:10.3762/bjoc.8.59)
Supplement: File 1 — Experimental procedures and spectroscopic data. [file Beilstein_J_Org_Chem-08-522-s001.pdf]

Supporting Information

for

Branching out at *C*-2 of septanosides. Synthesis of 2-deoxy-2-*C*-alkyl/aryl septanosides from a bromo-oxepine

Supriya Dey and Narayanaswamy Jayaraman*

Address: Department of Organic Chemistry, Indian Institute of Science, Bangalore 560 012, India

Email: Narayanaswamy Jayaraman* - jayaraman@orgchem.iisc.ernet.in

*Corresponding author

Experimental procedures and spectroscopic data

Contents

General methods S2

Syntheses of **3** and **5** S4

Synthesis of **6**...S5

Synthesis of **8**...S6

Synthesis of **10**...S6

Syntheses of **11** S7

Synthesis of **14**...S8

Figures 1 and 2. ^{1}H and ^{13}C NMR spectra of **3**...S9

Figures 3 and 4. ^{1}H and ^{13}C NMR spectra of **4**..S10

Figures 5 and 6. ^{1}H and ^{13}C NMR spectra of **5**......S11

Figures 7 and 8. ^{1}H and ^{13}C NMR spectra of **6**......S12

Figures 9 and 10. ^{1}H and ^{13}C NMR spectra of **7**......S13

Figures 11 and 12. ^{1}H and ^{13}C NMR spectra of **8**......S14

Figures 13 and 14. ^{1}H and ^{13}C NMR spectra of **9**......S15

Figures 15 and 16. ^{1}H and ^{13}C NMR spectra of **10**......S16

Figures 17 and 18. ^{1}H and ^{13}C NMR spectra of **11**......S17

Figures 19 and 20. ^{1}H and ^{13}C NMR spectra of **12**......S18

Figures 21 and 22. ^{1}H and ^{13}C NMR spectra of **13**......S19

Figures 23 and 24. ^{1}H and ^{13}C NMR spectra of **14**......S20

Figures 25 and 26. HSQC and COSY spectra of **5**......S21

Figures 27 and 28. COSY and HSQC spectra of **7**......S22

Figures 29 and 30. COSY and HSQC spectra of **9**......S23

Figures 31 and 32. COSY and HSQC spectra of **12**......S24

Figures 33 and 34. COSY and HSQC spectra of **13**......S25

Figures 35 and 36. COSY and HSQC spectra of **14**......S26

References......S27

General methods

Chemicals were purchased from commercial sources and used without further purification. Solvents were dried and distilled by following literature procedures. Bromo-oxepine was synthesized according to the reported procedure [1]. Analytical TLC was performed on commercial Merck plates coated with silica gel GF_{254} (0.25 mm) with detection by fluorescence and/or charring following immersion in 5% H_2SO_4/EtOH. Silica gel (230–400 mesh) was used for column chromatography. Optical rotations were recorded on a

polarimeter at the sodium D line at 24 °C. High resolution mass spectra were obtained from a Micromass Q-TOF instrument by the electrospray ionization (ESI) technique. ^{1}H and ^{13}C NMR spectral analyses were performed on 400 and 100 MHz NMR spectrometers, respectively, with the residual solvent signal acting as the internal standard. Standard abbreviations s, d, t, dd, br, app., m and band refer to singlet, doublet, triplet, doublet of doublet, broad, apparent, multiplet and set of resonances.

Experimental

Methyl 2-deoxy-2-*C*-(2-(methoxycarbonyl)ethyl)-3,4,5,7-tetra-*O*-benzyl-α-D-*arabino*-hept-2-enoseptanoside (**3**). A solution of **2** (0.05 g, 0.07 mmol) in 1,4-dioxane (1 mL) was admixed with $Pd(OAc)_2$ (1 mg, 10 mol %) under a N_2 atmosphere, and was followed by addition of Cs_2CO_3 (0.03 g, 0.11 mmol) and methyl acrylate (8 µL, 0.13 mmol), in a sealed tube. The reaction mixture was stirred at 98 °C for 72 h, cooled, filtered, diluted with EtOAc (20 mL), washed with water (2 × 30 mL) and brine (2 × 10 mL), dried (Na_2SO_4) and concentrated in vacuo. The resulting residue was purified (hexane/EtOAc 8:2) to afford **3** (0.035 g, 70%), as an oil.

R_f 0.30 (hexane/EtOAc 8:2); $[\alpha]_D$ −21.6 (*c* 0.5, $CHCl_3$); ^{1}H NMR (400 MHz, $CDCl_3$) δ 7.80 (d, *J* = 16.0 Hz, 1 H, C*H*=$CHCO_2$Me), 7.37–7.24 (m, 18 H, aromatic), 7.10–7.08 (m, 2 H, aromatic), 5.97 (d, *J* = 16.0 Hz, 1 H, CH=C*H*CO_2Me), 5.36 (s, 1 H, H-1), 4.67 (d, *J* = 4.0 Hz, 1 H, PhCH_2), 4.65 (d, *J* = 12.0 Hz, 1 H, PhCH_2), 4.59 (d, *J* = 12.0 Hz, 2 H, PhCH_2), 4.47 (d, *J* = 12.4 Hz, 1 H, PhCH_2), 4.43 (d, *J* = 12.0 Hz, 1 H, PhCH_2), 4.29 (d, *J* = 11.6 Hz, 2 H, PhCH_2), 4.20–4.15 (band, 2 H, H-4 and H-6), 3.77–3.75 (dd, *J* = 8.4, 2.0 Hz, 1 H, H-5), 3.73 (s, 3 H, CO_2Me), 3.60 (dd, *J* = 10.8, 6.0 Hz, 1 H, H-7a), 3.52–3.49 (band, 4 H, H-7b, OMe); ^{13}C NMR (100 MHz, $CDCl_3$) δ 167.8 (C=O), 159.5 (C-3), 138.5–137.2 (aromatic), 136.3 (*C*H=$CHCO_2$Me), 128.5–127.5 (aromatic), 124.2 (C-2), 119.5

($CH=CHCO_2Me$), 100.1 (C-1), 80.0 (C-5), 77.2 (C-4), 73.1 ($PhCH_2$), 72.9 ($PhCH_2$), 72.1 ($PhCH_2$), 71.2 ($PhCH_2$), 71.1 (C-6), 70.9 (C-7), 55.4 (OMe), 51.4 (CO_2Me); HRMS–ESI (*m*/*z*): $[M + Na]^+$ calcd for 673.2777; found, 673.2776.

Methyl 2-deoxy-2-*C*-((3-((5-(acryloyloxy)pentyl)oxy)-3-oxoprop-1-en-1-yl)-3,4,5,7-*tetra*-*O*-benzyl-α-D-*arabino*-hept-2-enoseptanoside (5). A solution of **2** (0.05 g, 0.07 mmol) in 1,4-dioxane (1 mL) was admixed with $Pd(OAc)_2$ (1 mg, 10 mol %) under a N_2 atmosphere, and was followed by addition of Cs_2CO_3 (0.03 g, 0.11 mmol) and $H_2C=CH\text{-}COO(CH_2)_5OCOCH=CH_2$ (0.01 g, 0.04 mmol), in a sealed tube. The reaction mixture was stirred at 98 °C for 72 h, cooled, filtered, diluted with EtOAc (20 mL), washed with water (2 × 30 mL) and brine (2 × 10 mL), dried (Na_2SO_4) and concentrated in vacuo. The resulting residue was purified (hexane/EtOAc 5.7:1) to afford **5** (0.034 g, 60%, based on **2**), as an oil.

R_f 0.20 (hexane/EtOAc 9:1); $[\alpha]_D$ −124.3 (*c* 0.1, $CHCl_3$); ^1H NMR (400 MHz, $CDCl_3$) δ 7.79 (d, *J* = 16.4 Hz, 1 H, $\text{-}CH=CHCOO(CH_2)_5\text{-}$), 7.33–7.18 (m, 18 H, aromatic), 7.10–7.08 (m, 2 H, aromatic), 6.39 (dd, *J* = 17.2, 1.2 Hz, 1 H, $\text{-}CH=CH_2$), 6.10 (dd, *J* = 17.2, 10.4 Hz, 1 H, $\text{-}CH=CH_2$), 5.93 (d, *J* = 16.4 Hz, 1 H, $\text{-}CH=CHCOO(CH_2)_5\text{-}$), 5.80 (dd, *J* = 10.4, 1.2 Hz, 1 H,$\text{-}CH=CH_2$), 5.36 (s, 1 H, H-1), 4.67 (d, *J* = 12.4 Hz, 2 H, $PhCH_2$), 4.57 (d, *J* = 11.6 Hz, 2 H, $PhCH_2$), 4.45 (m, 3 H, $PhCH_2$), 4.33 (d, *J* = 11.6 Hz, 1 H, $PhCH_2$), 4.20–4.10 (band, 6 H, H-4, H-6, $\text{-}OCH_2(CH_2)_3CH_2O\text{-}$), 3.76 (dd, *J* = 8.4, 2.0 Hz, 1 H, H-5), 3.58 (dd, *J* = 10.6, 6.2, 1 H, H-7a), 3.53–3.50 (band, 4 H, H-7b, OMe), 1.73–1.66 (m, 4 H, $\text{-}COOCH_2(CH_2)_3CH_2OCO\text{-}$), 1.48–1.41 (m, 2 H, $\text{-}COOCH_2\text{-}(CH_2)_3CH_2OCO\text{-}$); ^{13}C NMR (100 MHz, $CDCl_3$) δ 167.3 (C=O), 166.2 (C=O), 159.3 (C-3), 138.3 ($\text{-}CH=CHCOO(CH_2)_5\text{-}$), 138.1–136.3 (aromatic), 130.4 ($\text{-}COO(CH_2)_5OCO\text{-}CH=CH_2$), 128.5 ($\text{-}OCOCH=CH_2$), 128.4–127.5 (aromatic), 124.2 (C-2), 119.8

(-CH=*C*HCOO(CH_2)$_5$-), 100.0 (C-1), 80.0 (C-5), 76.6 (C-4), 73.0 (*C*H_2Ph), 72.9 (*C*H_2Ph), 72.1 (*C*H_2Ph), 71.2 (*C*H_2Ph), 71.1 (C-6), 70.9 (C-7), 64.3 (-COO*C*H_2(CH_2)$_3$$CH_2$OCO-), 63.9 (-COO$CH_2$($CH_2$)$_3$*C*$H_2$OCO-), 55.4 (OMe), 28.3 (-COOCH_2(*C*H_2)$_3$$CH_2$OCO-), 28.2 (-COO$CH_2$(*C*$H_2$)$_3$$CH_2$OCO-), 22.4 (-COO$CH_2$(*C*$H_2$)$_3$$CH_2$OCO-); HRMS–ESI (*m*/*z)*: $[M + Na]^+$ calcd for 799.92; found, 800.17.

Methyl 2-deoxy-2-*C*-(2-phenylethenyl)-3,4,5,7-*tetra*-*O*-benzyl-*α*-D-arabino-hept-2-enoseptanoside (**6**). A solution of **2** (0.05 g, 0.07 mmol) in 1,4-dioxane (1 mL) was admixed with $Pd(OAc)_2$ (1 mg, 10 mol %) under a N_2 atmosphere, and was followed by addition of Cs_2CO_3 (0.03 g, 0.11 mmol) and styrene (0.01 mL, 0.09 mmol), in a sealed tube. The reaction mixture was stirred at 98 °C for 72 h, cooled, filtered, diluted with EtOAc (20 mL), washed with water (2 × 30 mL) and brine (2 × 10 mL), dried (Na_2SO_4) and concentrated in vacuo. The resulting residue was purified (hexane/EtOAc 9:1) to afford **6** (0.043 g, 74%), as an oil.

R_f 0.29 (hexane/EtOAc 9:1); $[\alpha]_D$ −54.6 (*c* 0.1, $CHCl_3$); ^{1}H NMR (400 MHz, $CDCl_3$) δ 7.37–7.23 (m, 23 H, aromatic), 7.19 (d, *J* = 16.8 Hz, 1 H, -C*H*=CHPh), 7.12–7.09 (m, 2 H, aromatic), 6.66 (d, *J* = 16.8 Hz, 1 H, -CH=C*H*Ph), 5.48 (s, 1 H, H-1), 4.72 (d, *J* = 12.8 Hz, 1 H, PhC*H*$_2$), 4.67–4.57 (m, 3 H, PhC*H*$_2$), 4.50 (d, *J* = 12.8 Hz, 2 H, PhC*H*$_2$), 4.38 (d, *J* = 12.0 Hz, 1 H, PhC*H*$_2$), 4.29–4.22 (band, 3 H, H-4, H-6 and PhC*H*$_2$), 3.76 (dd, *J* = 8.4, 2.4 Hz, 1 H, H-5), 3.65 (dd, *J* = 10.4, 6.4 Hz, 1 H, H-7a), 3.58 (s, 3 H, OMe), 3.54 (dd, *J* = 10.4, 2.8 Hz, 1 H, H-7b); ^{13}C NMR (100 MHz, $CDCl_3$) δ 154.2 (C-3), 138.3–137.1 (aromatic), 130.6 (-*C*H=CHPh), 128.4–127.3 (aromatic), 126.4 (C-2), 122.9 (-CH=*C*HPh), 100.6 (C-1), 80.3 (C-5), 77.8 (C-4), 72.9 (Ph*C*H_2), 72.8 (2 × Ph*C*H_2), 71.9 (Ph*C*H_2), 71.1 (C-6), 71.0 (C-7), 55.4 (OMe); HRMS–ESI (*m*/*z)*: $[M + Na]^+$ calcd for 691.3036; found, 691.3047.

Methyl 2-deoxy-2-*C*-phenyl-3,4,5,7-*tetra*-*O*-benzyl-α-D-*arabino*-hept-2-enoseptanoside (8). A solution of **2** (0.05 g, 0.07 mmol) in 1,4-dioxane (1 mL) was admixed with $Pd(OAc)_2$ (1 mg, 10 mol %) under a N_2 atmosphere, and was followed by addition of Cs_2CO_3 (0.03 g, 0.11 mmol) and phenylboronic acid (0.02 g, 0.15 mmol), in a sealed tube. The reaction mixture was stirred at 98 °C for 72 h, cooled, filtered, diluted with EtOAc (20 mL), washed with water (2 × 30 mL) and brine (2 × 10 mL), dried (Na_2SO_4) and concentrated in vacuo. The crude reaction mixture was purified (hexane/EtOAc 9:1) to afford **8** (0.038 g, 79 %), as a gum.

R_f 0.45 (hexane/EtOAc 9:1); $[\alpha]_D$ −14.2 (*c* 0.1, $CHCl_3$); ^{1}H NMR (400 MHz, $CDCl_3$) δ 7.38–7.12 (m, 23 H, aromatic), 6.83 (d, *J* = 6.0 Hz, 2 H, aromatic), 5.41 (s, 1 H, H-1), 4.82 (d, *J* = 12.0 Hz, 1 H, $PhCH_2$), 4.63 (d, *J* = 12.0 Hz, 1 H, $PhCH_2$), 4.57 (d, *J* = 12.4 Hz, 2 H, $PhCH_2$), 4.50 (d, *J* = 12.0 Hz, 1 H, $PhCH_2$), 4.39 (d, *J* = 11.2 Hz, 2 H, $PhCH_2$), 4.34–4.31 (m, 1 H, H-6), 4.28 (app. d, *J* = 12.0 Hz, 1 H, H-4), 4.24 (d, *J* = 11.6 Hz, 1 H, $PhCH_2$), 3.80 (dd, *J* = 8.4, 1.6 Hz, 1 H, H-5), 3.66 (dd, *J* = 10.4, 6.0 Hz, 1 H, H-7a), 3.58 (dd, *J* = 10.4, 2.4 Hz, 1 H, H-7b), 3.31 (s, 3 H, OMe); ^{13}C NMR (100 MHz, $CDCl_3$) δ 152.3 (C-3), 138.4–136.8 (aromatic), 129.6 (aromatic), 129.0 (C-2), 128.3–126.7 (aromatic), 102.0 (C-1), 80.6 (C-5), 77.9 (C-4), 73.0 ($PhCH_2$), 72.6 ($PhCH_2$), 72.0 ($PhCH_2$), 71.2 ($PhCH_2$), 71.1 (C-6), 71.0 (C-7), 55.9 (OMe); HRMS–ESI (*m/z*): $[M + Na]^+$ calcd for 665.2879; found, 665.2878.

Methyl 2-deoxy-2-*C*-(*m*-tolyl)-3,4,5,7-tetra-*O*-benzyl-α-D-*arabino*-hept-2-enoseptano-side (10). A solution of **2** (0.05 g, 0.07 mmol) in 1,4-dioxane (1 mL) was admixed with $Pd(OAc)_2$ (1 mg, 10 mol %) under a N_2 atmosphere, and was followed by addition of Cs_2CO_3 (0.03 g, 0.11 mmol) and 3-methylphenylboronic acid (0.010 g, 0.07 mmol), in a sealed tube. The reaction mixture was stirred at 98 °C for 72 h, cooled, filtered, diluted

with EtOAc (20 mL), washed with water (2 × 30 mL) and brine (2 × 10 mL), dried (Na_2SO_4) and concentrated in vacuo. The crude reaction mixture was purified (hexane/EtOAc 9:1) to afford **10** (0.032 g, 61%), as an oil.

R_f 0.40 (hexane/EtOAc 9:1); $[\alpha]_D$ +10.0 (*c* 0.5, $CHCl_3$); ^1H NMR (400 MHz, $CDCl_3$) δ 7.38–7.14 (m, 20 H, aromatic), 7.05–6.99 (m, 2 H, aromatic), 6.85 (d, *J* = 6.8 Hz, 2 H, aromatic), 5.41 (s, 1 H, H-1), 4.80 (d, *J* = 12.4 Hz, 1 H, PhCH_2), 4.62 (d, *J* = 12.4 Hz, 1 H, PhCH_2), 4.56 (d, *J* = 12.4 Hz, 1 H, PhCH_2), 4.50 (d, *J* = 12.4 Hz, 1 H, PhCH_2), 4.40 (d, *J* = 9.6 Hz, 2 H, PhCH_2), 4.32–4.27 (m, 2 H, H-4, H-6), 4.25 (d, *J* = 11.6 Hz, 2 H, PhCH_2), 3.80 (dd, *J* = 8.8, 2.0 Hz, 1 H, H-5), 3.66 (dd, *J* = 10.4, 6.0 Hz, 1 H, H-7a), 3.58 (dd, *J* = 10.4, 2.4 Hz, 1 H, H-7b), 3.31 (s, 3 H, OMe), 2.29 (s, 3 H, Me); ^{13}C NMR (100 MHz, $CDCl_3$) δ 152.0 (C-3), 138.4–136.6 (aromatic), 130.2 (aromatic), 129.2 (C-2), 128.9–126.4 (aromatic), 102.1 (C-1), 80.6 (C-5), 78.0 (C-4), 73.0 (Ph*C*H_2), 72.6 (Ph*C*H_2), 72.1 (Ph*C*H_2), 71.2 (C-6), 71.1 (C-7), 55.8 (OMe), 21.4 (Me); ESI-MS *m*/*z* $[M + Na]^+$ calcd for 679.3036; found, 679.3016.

Methyl 2-deoxy-2-*C*-(2-phenylethynyl)-3,4,5,7-*tetra*-*O*-benzyl-α-D-*arabino*-hept-2-enoseptanoside (11). A solution of **2** (0.05 g, 0.07 mmol) in DMF/THF/Et_3N 5:3:2 (1 mL) was admixed with $Pd(PPh_3)_2Cl_2$ (0.01 g, 20 mol %) under N_2 atmosphere, and was followed by addition of CuI (0.012 g, 10 mol %) and phenylacetylene (0.017 mL, 0.15 mmol), in a sealed tube. The reaction mixture was stirred at 98 °C for 72 h, cooled, filtered, diluted with EtOAc (20 mL), washed with water (2 × 30 mL) and brine (2 × 10 mL), dried (Na_2SO_4) and concentrated in vacuo. The crude reaction mixture was purified (hexane/EtOAc 9:1) to afford **11** (0.039 g, 77%), as a semi-solid.

R_f 0.45 (hexane/EtOAc 9:1); $[\alpha]_D$ +22.0 (*c* 0.1, $CHCl_3$); ^1H NMR (400 MHz, $CDCl_3$) δ 7.59–7.23 (m, 23 H, aromatic), 7.08–7.06 (m, 2 H, aromatic), 5.39 (s, 1 H, H-1), 5.19 (d,

J = 11.6 Hz, 1 H, PhC*H*$_2$), 4.93 (d, *J* = 11.6 Hz, 1 H, PhC*H*$_2$), 4.75 (d, *J* = 12.2 Hz, 1 H, PhC*H*$_2$), 4.61 (d, *J* = 12.2 Hz, 1 H, PhC*H*$_2$), 4.48 (d, *J* = 11.8, 2 H, PhC*H*$_2$), 4.37 (d, *J* = 11.8 Hz, 1 H, PhC*H*$_2$), 4.33–4.22 (m, 3 H, H-4, H-6 and PhC*H*$_2$), 3.73 (app. d, *J* = 9.2 Hz, 1 H, H-5), 3.62 (dd, *J* = 10.4, 6.0 Hz, 1 H, H-7a), 3.53–3.49 (band, 4 H, H-7b, OMe); ^{13}C NMR (100 MHz, CDCl$_3$) δ 162.1 (C-3), 138.3–137.2 (aromatic), 131.3 (aromatic), 128.3–123.6 (aromatic), 108.4 (C-2), 100.5 (C-1), 95.8 (-*C*≡CPh), 85.2 (-C≡*C*Ph), 80.3 (C-5), 78.5 (C-4), 73.6 (Ph*C*H$_2$), 72.8 (Ph*C*H$_2$), 71.9 (Ph*C*H$_2$), 71.2 (Ph*C*H$_2$), 71.1 (C-6), 70.6 (C-7), 55.9 (OMe); HRMS–ESI (*m*/*z*): [M + Na]$^+$ calcd for 689.2879; found, 689.2877.

Methyl 2-deoxy-2-*C*-(2-(*tert*-butoxycarbonyl)ethyl)-α-D-*glycero*-D-*ido*-septanoside (14). To a solution of **13** (0.017 g, 0.05 mmol) in MeOH (2 mL) was added NaBH$_4$ (0.003 g, 0.07 mmol) at 0 °C and the mixture was stirred for 3 h at rt. The solvents were then removed in vacuo, and the resulting residue was dissolved in EtOAc (2 × 15 mL), washed with brine (10 mL), dried (Na$_2$SO$_4$), concentrated in vacuo and purified (CHCl$_3$/MeOH 8 :2) to afford **14** (0.016 g, 93%), as a colorless oil.

R_f 0.3 (MeOH/CHCl$_3$ 8:2); $[\alpha]_D$ +21.5 (*c* 0.5, MeOH); ^{1}H NMR (400 MHz, D$_2$O) δ 4.50 (d, *J* = 6.8 Hz, 1 H, H-1), 4.24 (band, 2 H, H-6, H-7a), 3.77 (dd, *J* = 12.4, 4.8 Hz, 1 H, H-7b), 3.73–3.63 (app. ddd, *J* = 12.4, 9.6, 4. 8 Hz, 2 H, H-3, H-4), 3.49 (s, 3 H, OMe), 3.47–3.44 (br, 1 H, H-5), 2.47–2.34 (br, 2 H, -CH$_2$C*H*$_2$CO$_2$*t*-Bu), 1.89–1.78 (m, 3 H, H-2, -C*H*$_2$CH$_2$CO$_2$*t*-Bu), 1.49 (s, 9 H, *t*-Bu); ^{13}C NMR (100 MHz, D$_2$O) δ 175.4 (C=O), 103.7 (C-1), 82.5 (*C*-*t*-Bu), 78.7 (C-5), 73.1 (C-6), 70.3 (C-4), 70.1 (C-3), 61.9 (C-7), 55.4 (OMe), 42.6 (C-2), 33.0 (-CH$_2$*C*H$_2$CO$_2$*t*-Bu), 27.2 (*t*-Bu), 25.7 (-*C*H$_2$CH$_2$CO$_2$*t*-Bu); HRMS–ESI (*m*/*z*): [M + Na]$^+$ calcd for 359.1682; found, 359.1680.

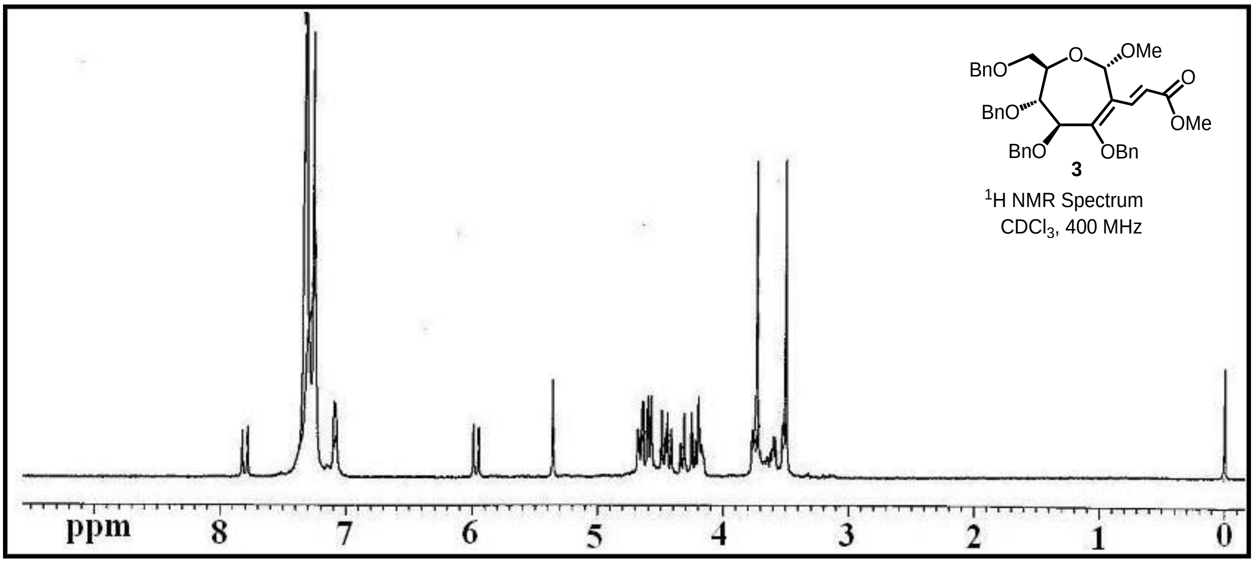

Figure 1: ^{1}H NMR spectrum of **3** (400 MHz, $CDCl_3$).

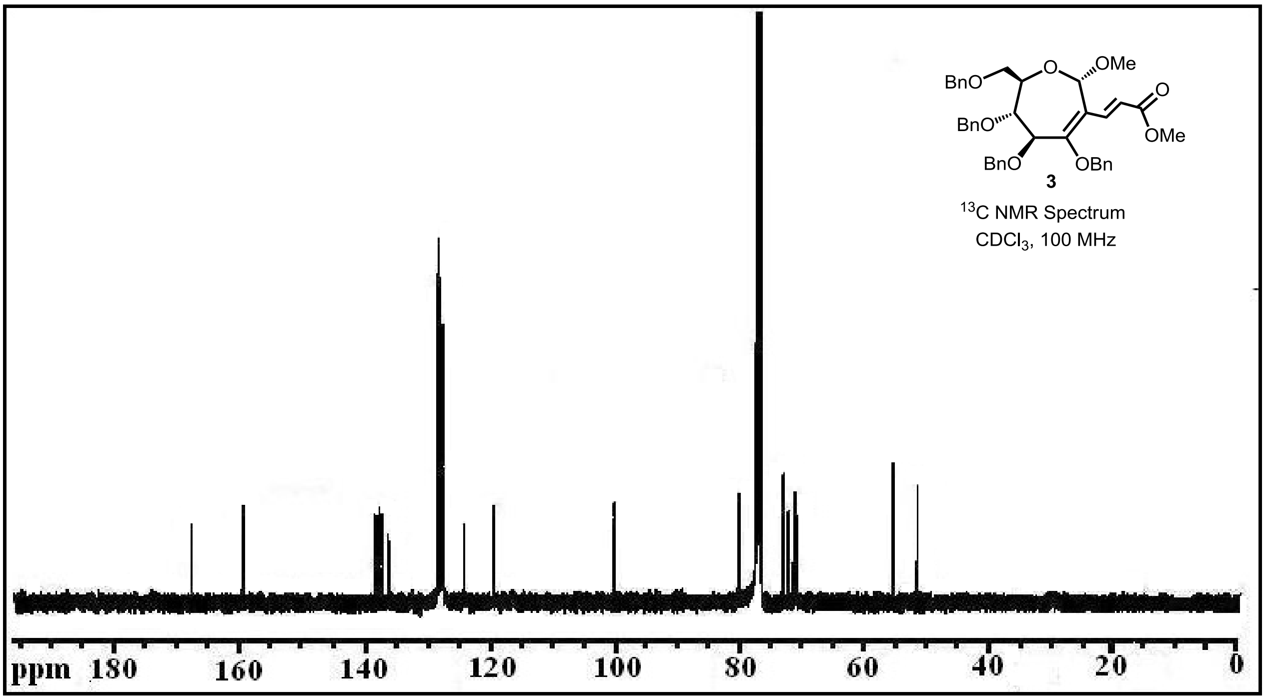

Figure 2: ^{13}C NMR spectrum of **3** (100 MHz, $CDCl_3$).

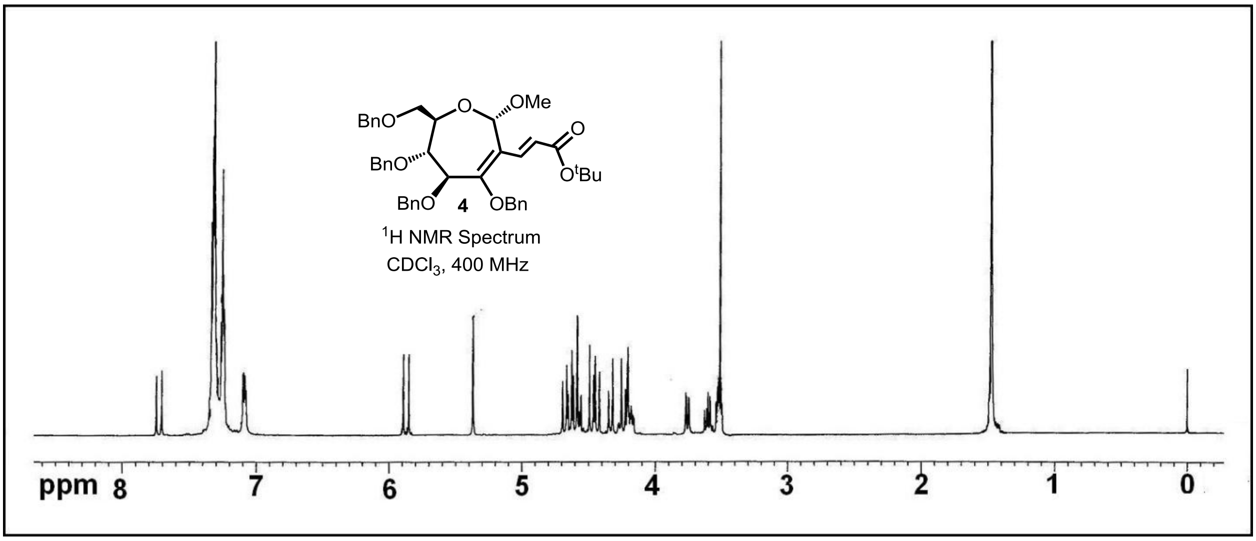

Figure 3: ^{1}H NMR spectrum of **4** (400 MHz, $CDCl_3$).

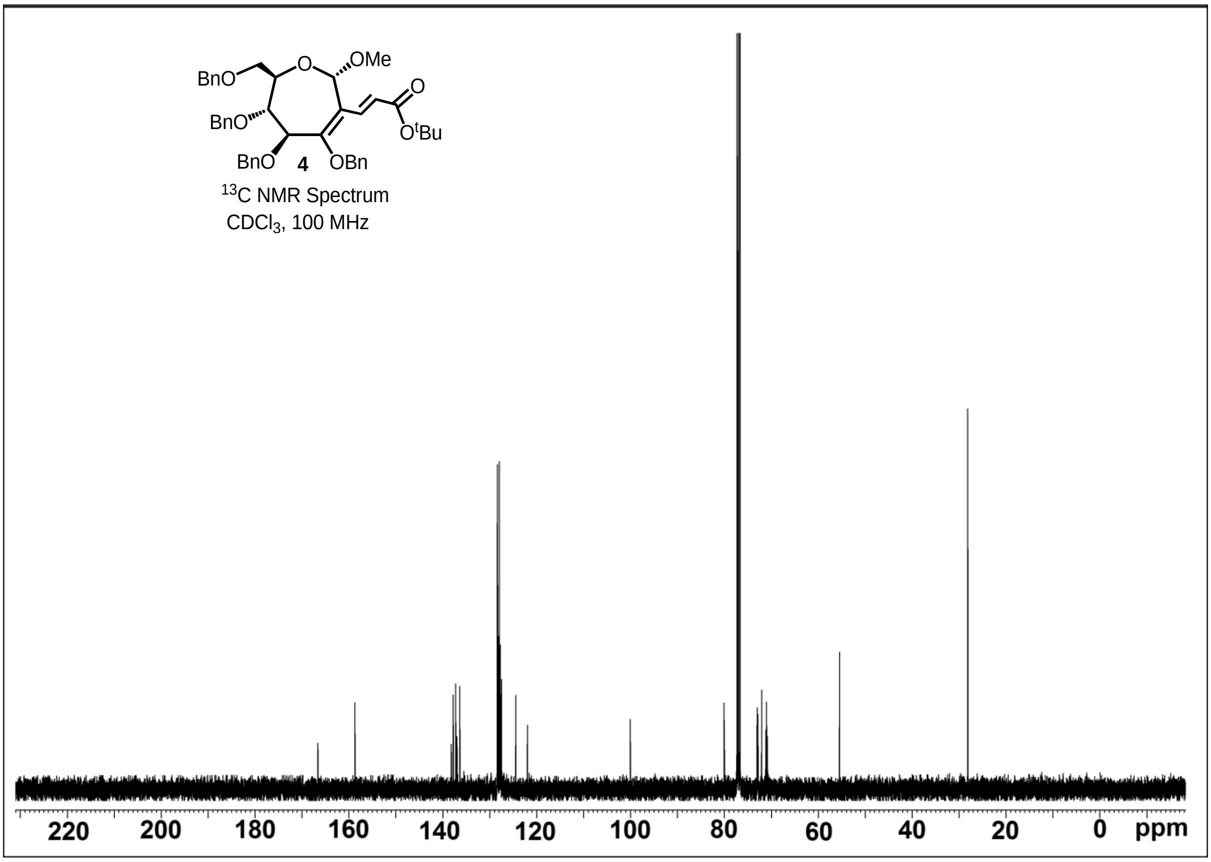

Figure 4: ^{13}C NMR spectrum of **4** (100 MHz, $CDCl_3$).

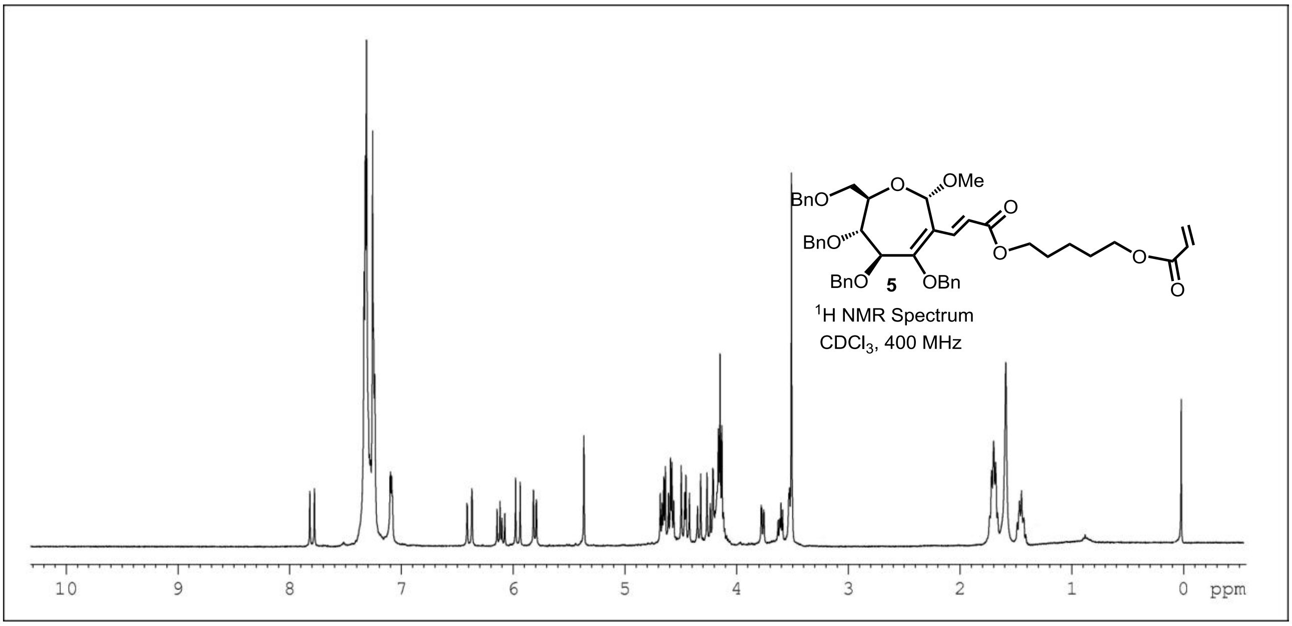

Figure 5: ^{1}H NMR spectrum of **5** (400 MHz, $CDCl_3$).

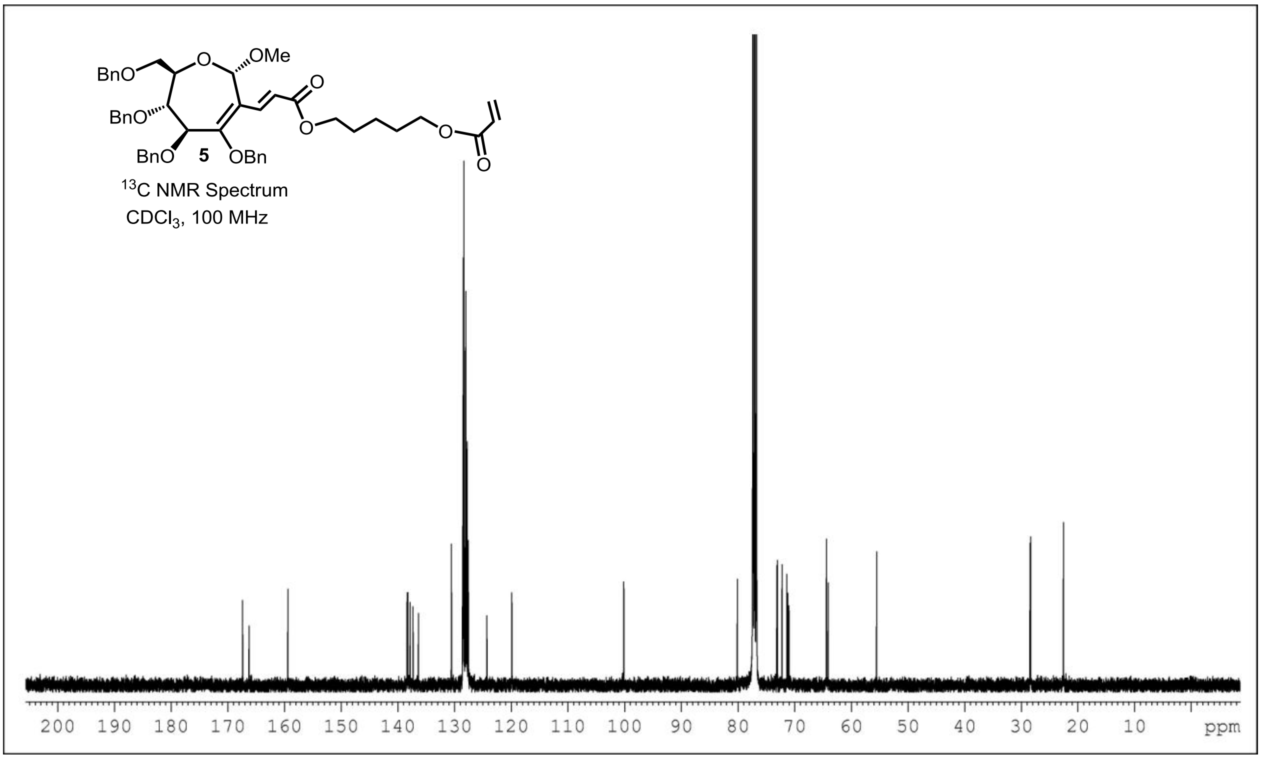

Figure 6: ^{13}C NMR spectrum of **5** (100 MHz, $CDCl_3$).

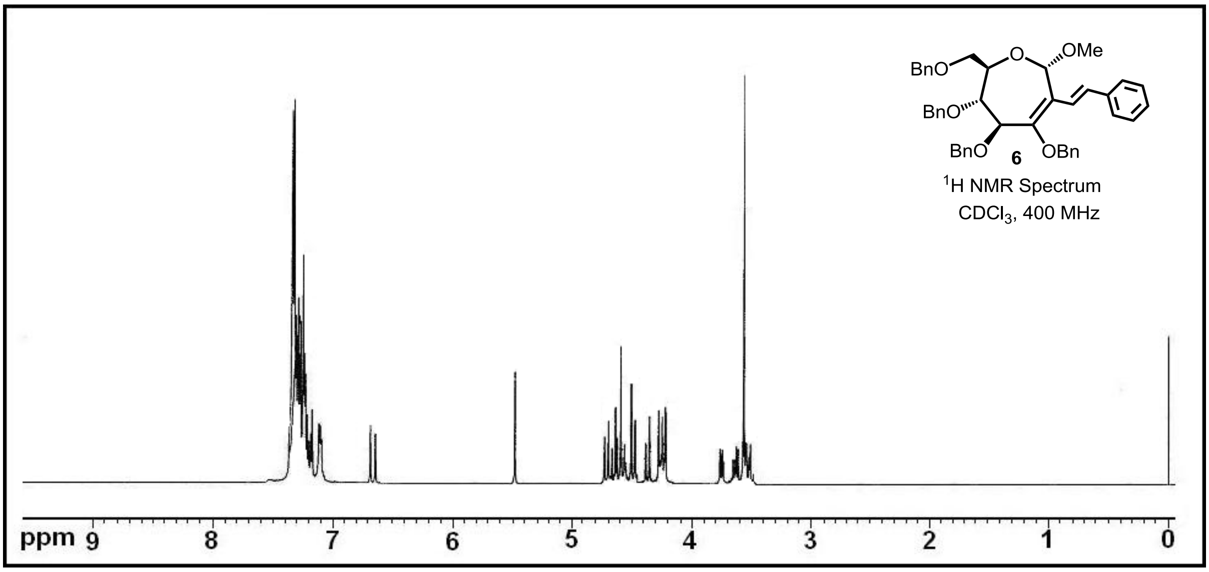

Figure 7: ^{1}H NMR spectrum of **6** (400 MHz, $CDCl_3$).

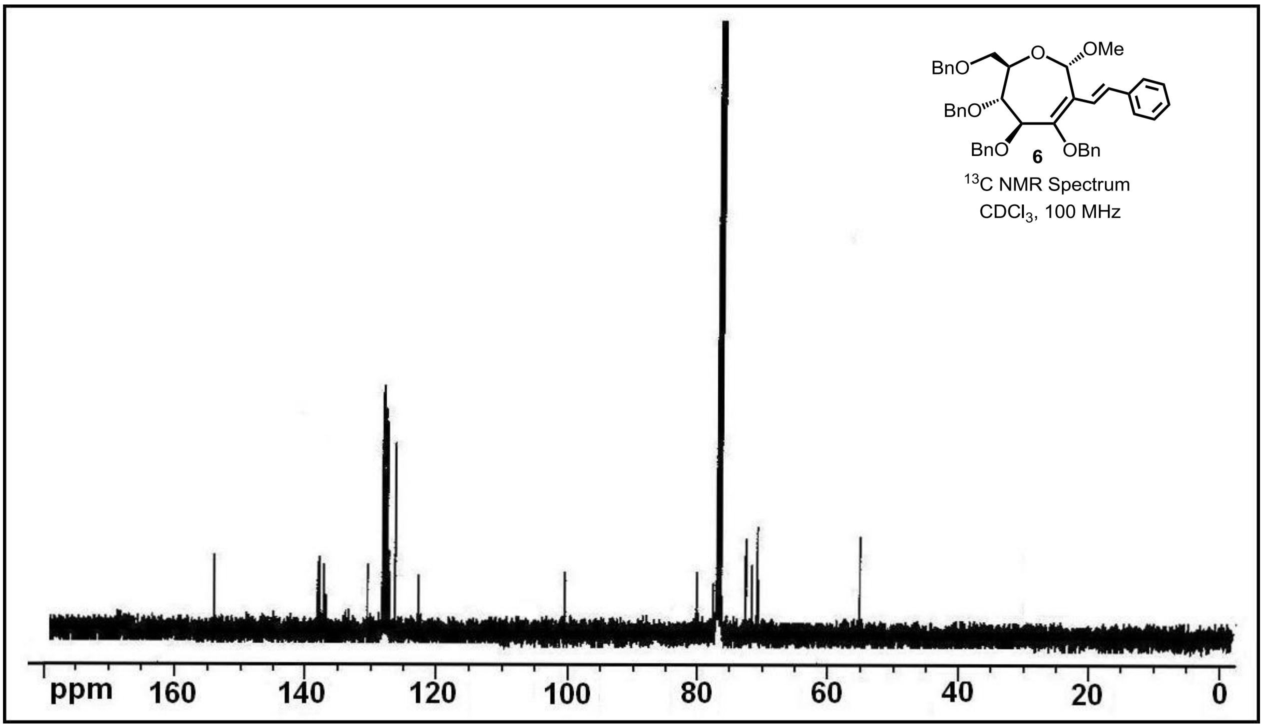

Figure 8: ^{13}C NMR spectrum of **6** (100 MHz, $CDCl_3$).

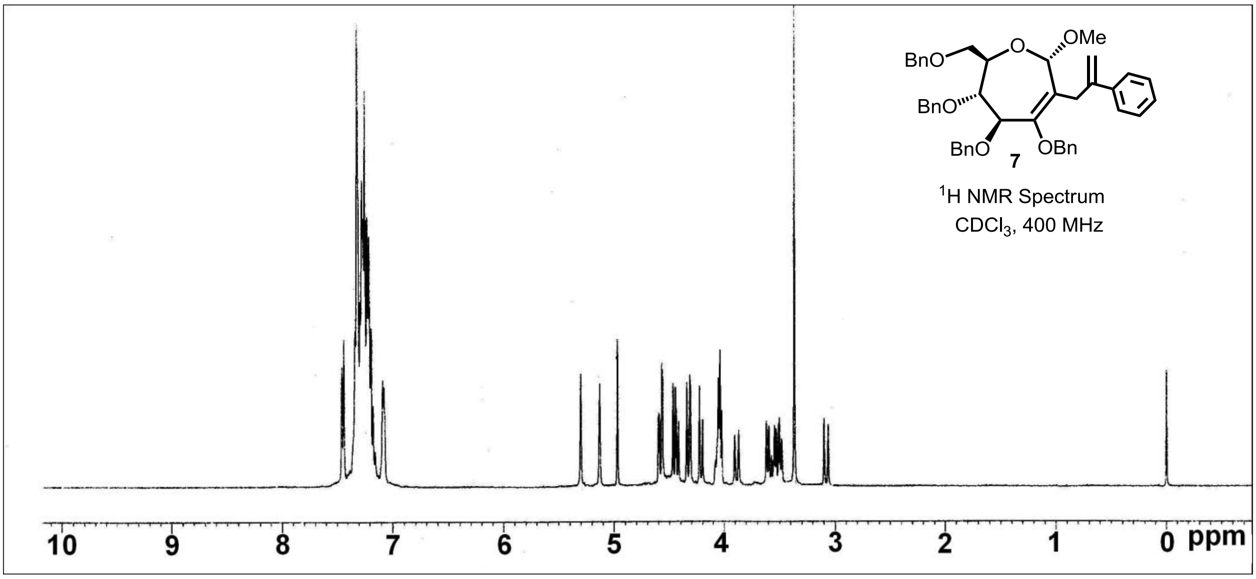

Figure 9: ^{1}H NMR spectrum of **7** (400 MHz, $CDCl_3$).

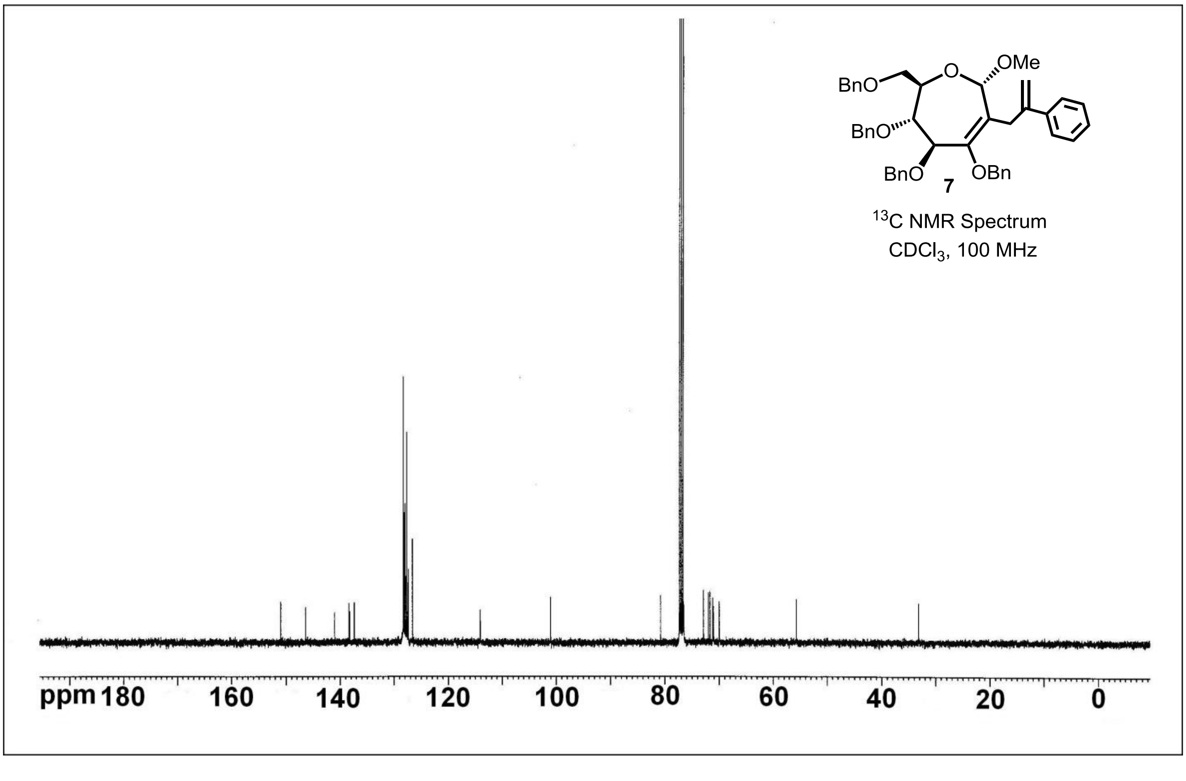

Figure 10: ^{13}C NMR spectrum of **7** (100 MHz, $CDCl_3$).

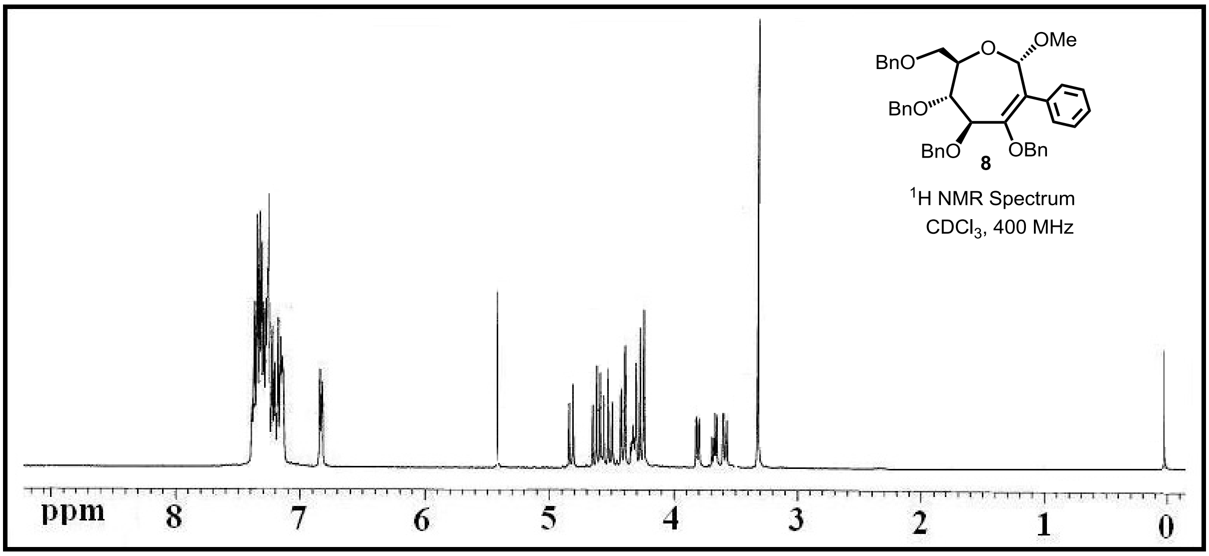

Figure 11: ^{1}H NMR spectrum of **8** (400 MHz, $CDCl_3$).

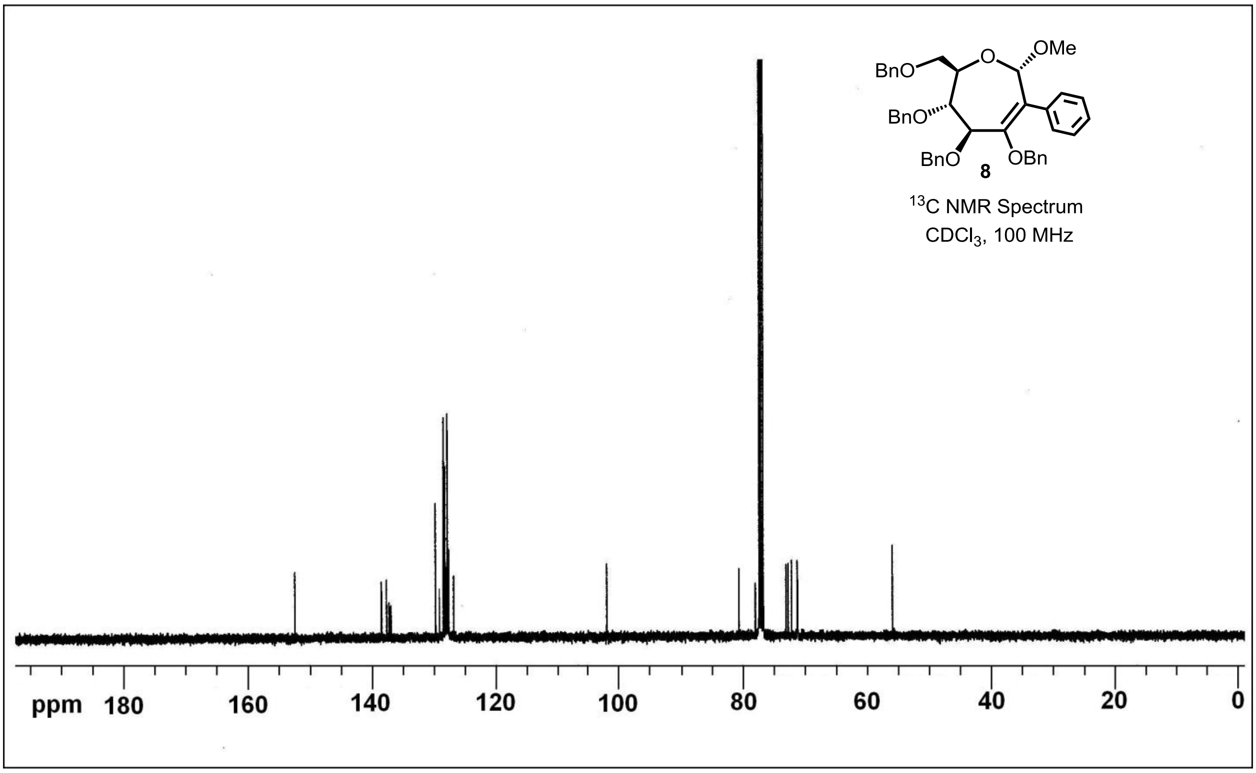

Figure 12: ^{13}C NMR spectrum of **8** (100 MHz, $CDCl_3$).

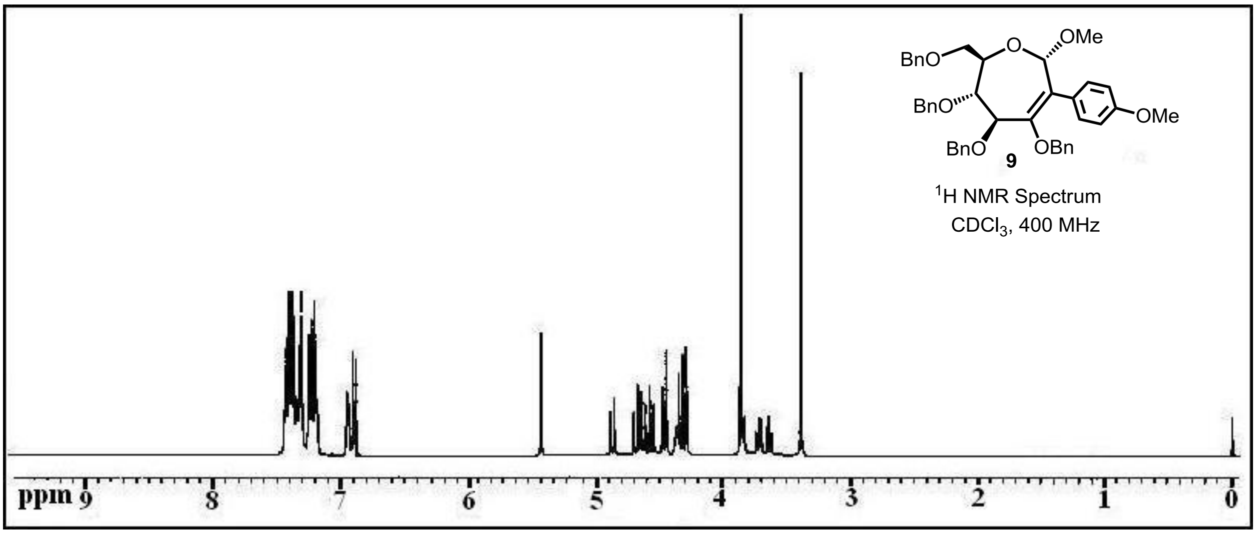

Figure 13: ^{1}H NMR spectrum of **9** (400 MHz, $CDCl_3$).

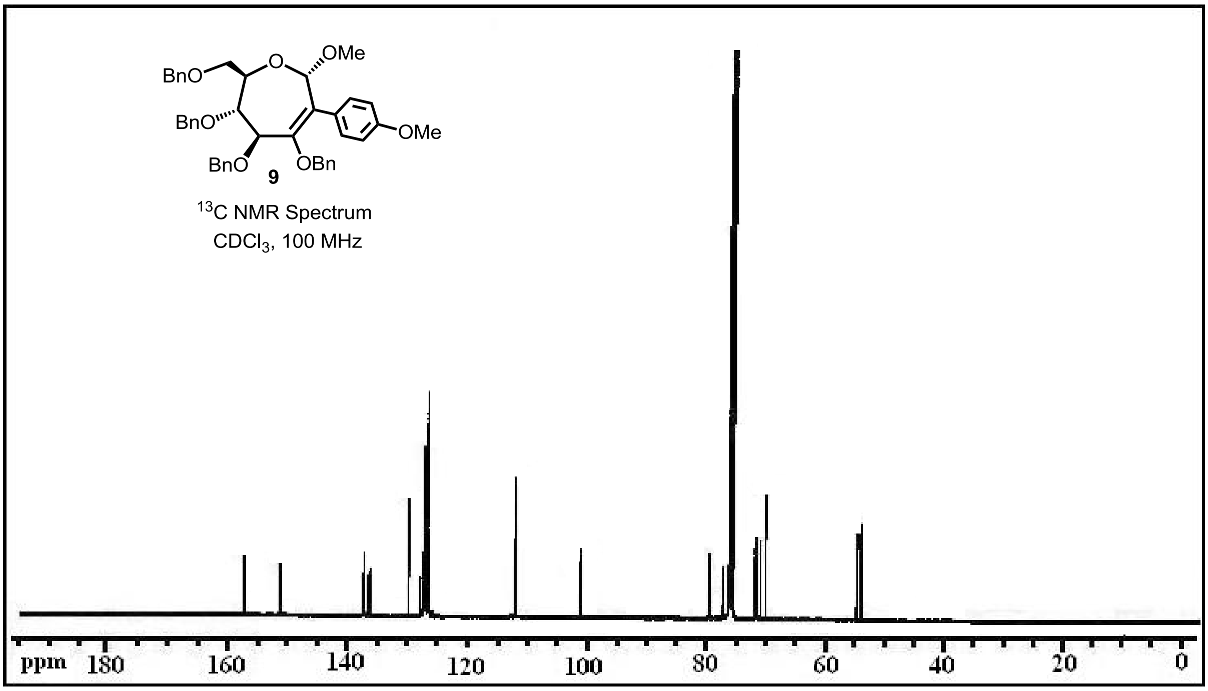

Figure 14: ^{13}C NMR spectrum of **9** (100 MHz, $CDCl_3$).

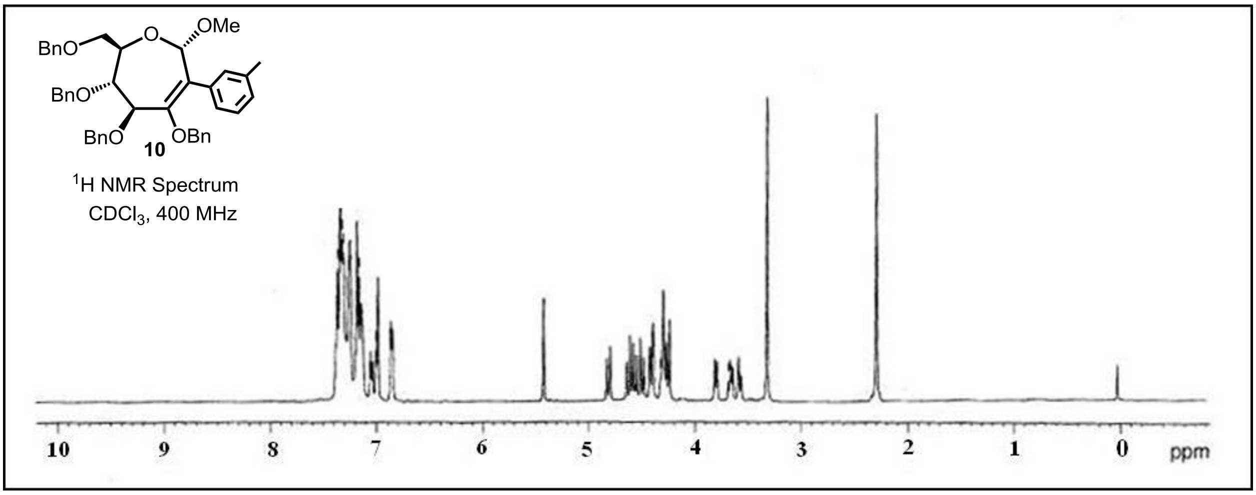

Figure 15: ^{1}H NMR spectrum of **10** (400 MHz, $CDCl_3$).

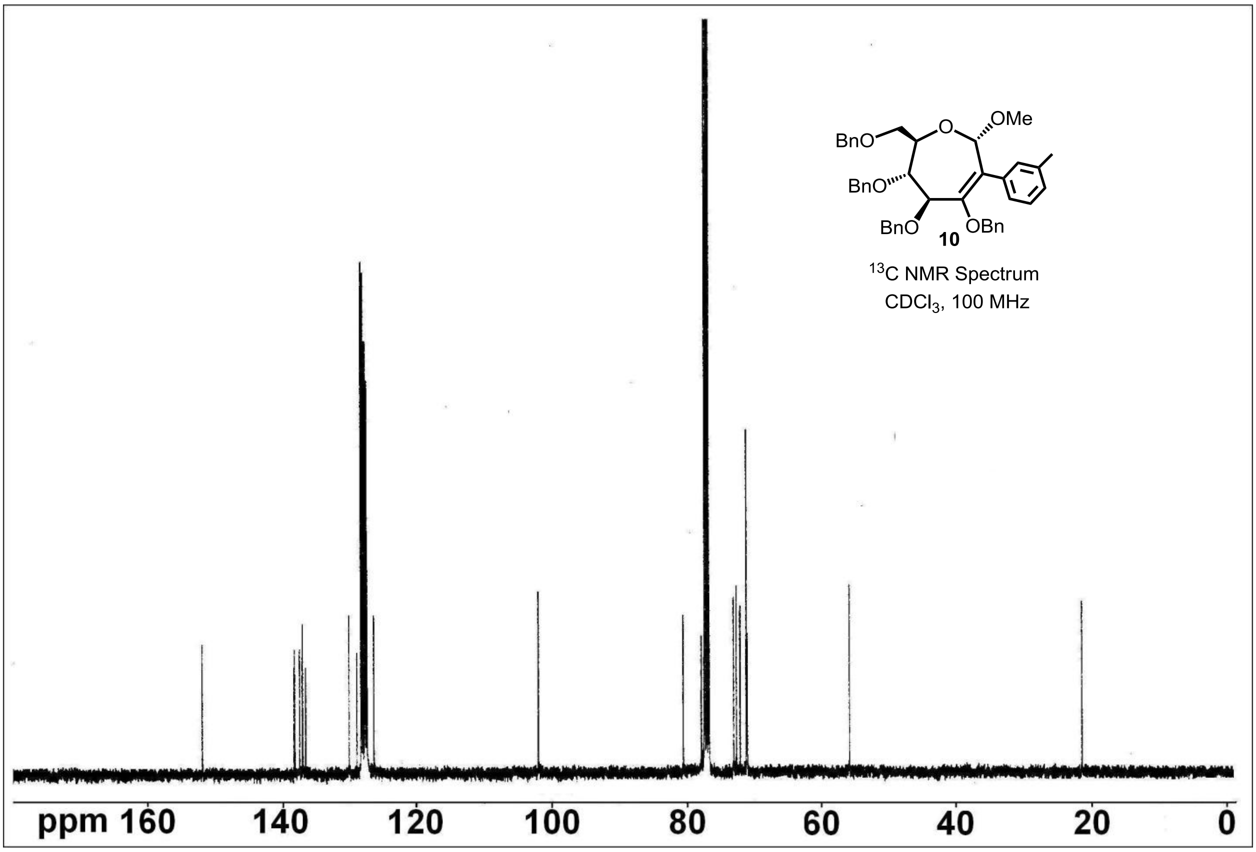

Figure 16: ^{13}C NMR spectrum of **10** (100 MHz, $CDCl_3$).

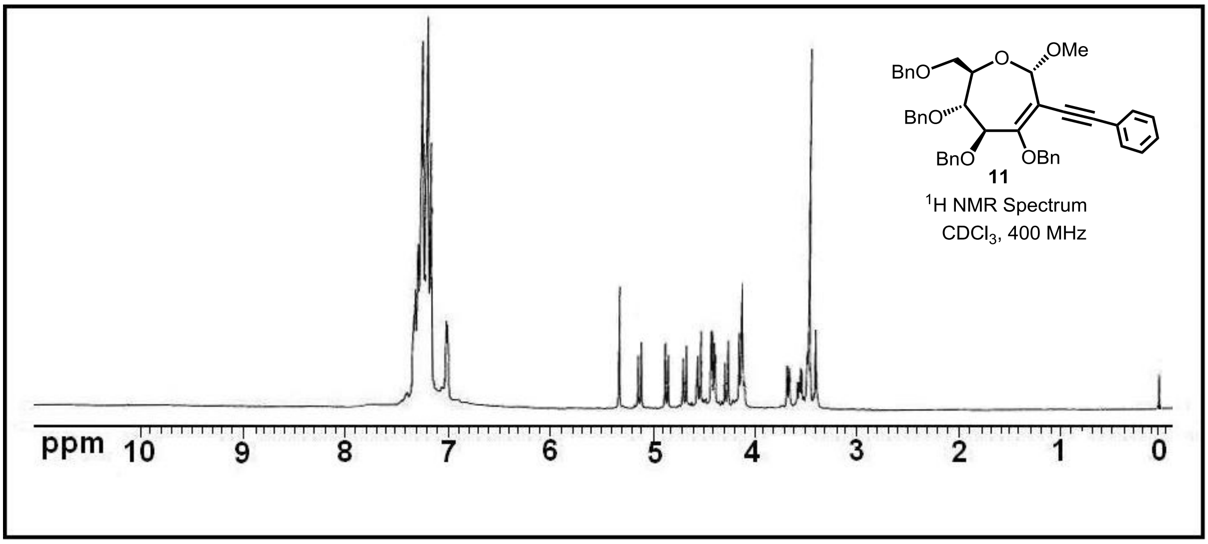

Figure 17: ^{1}H NMR spectrum of **11** (400 MHz, $CDCl_3$).

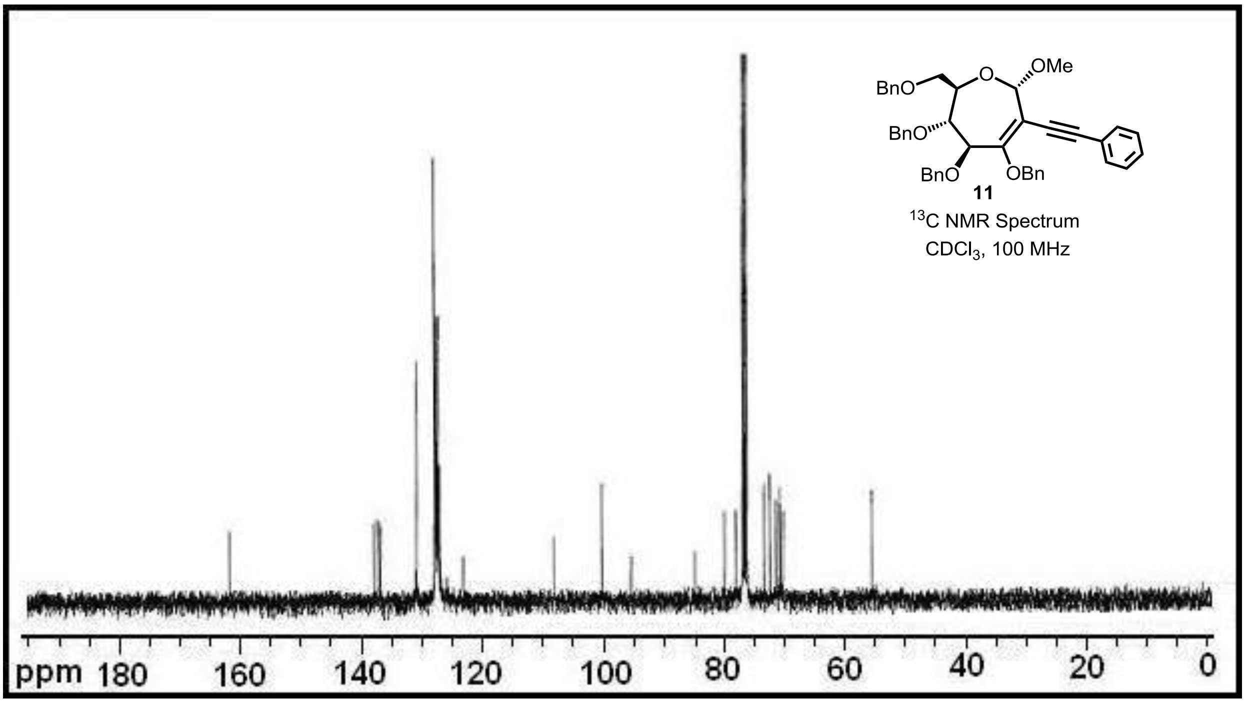

Figure 18: ^{13}C NMR spectrum of **11** (100 MHz, $CDCl_3$).

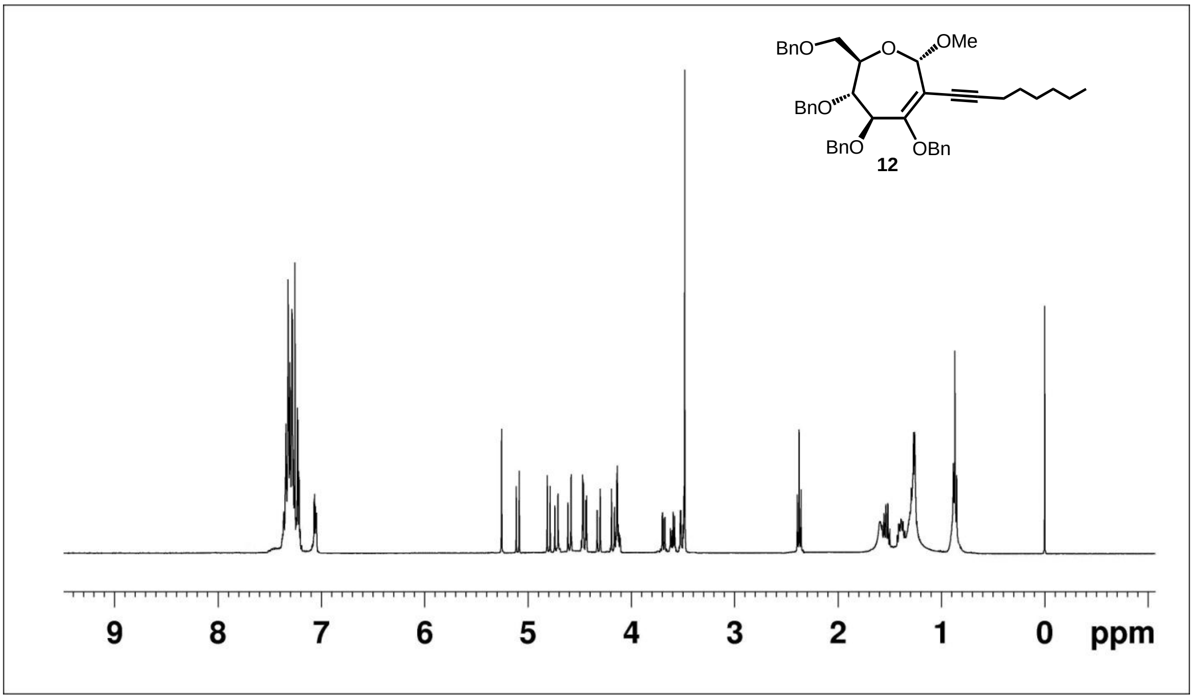

Figure 19: ^{1}H NMR spectrum of **12** (400 MHz, $CDCl_3$).

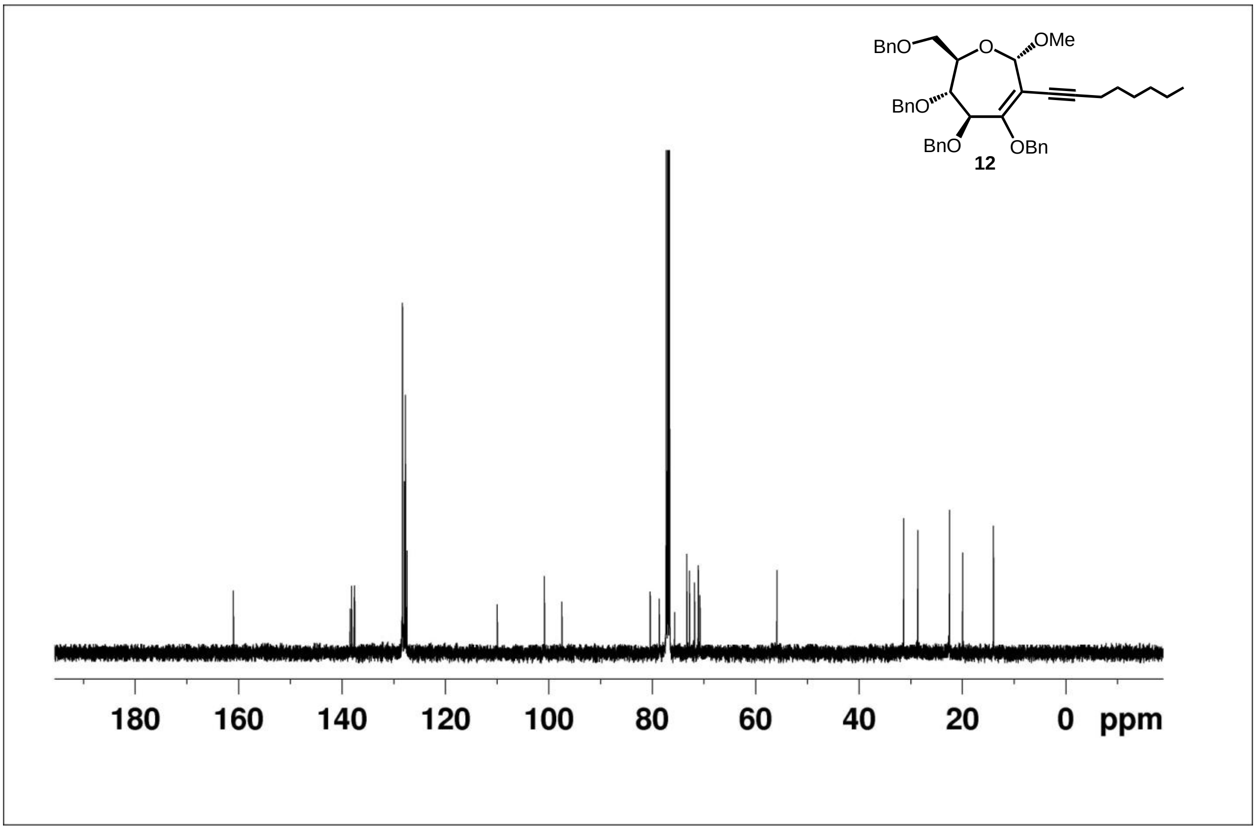

Figure 20: ^{13}C NMR spectrum of **12** (100 MHz, $CDCl_3$).

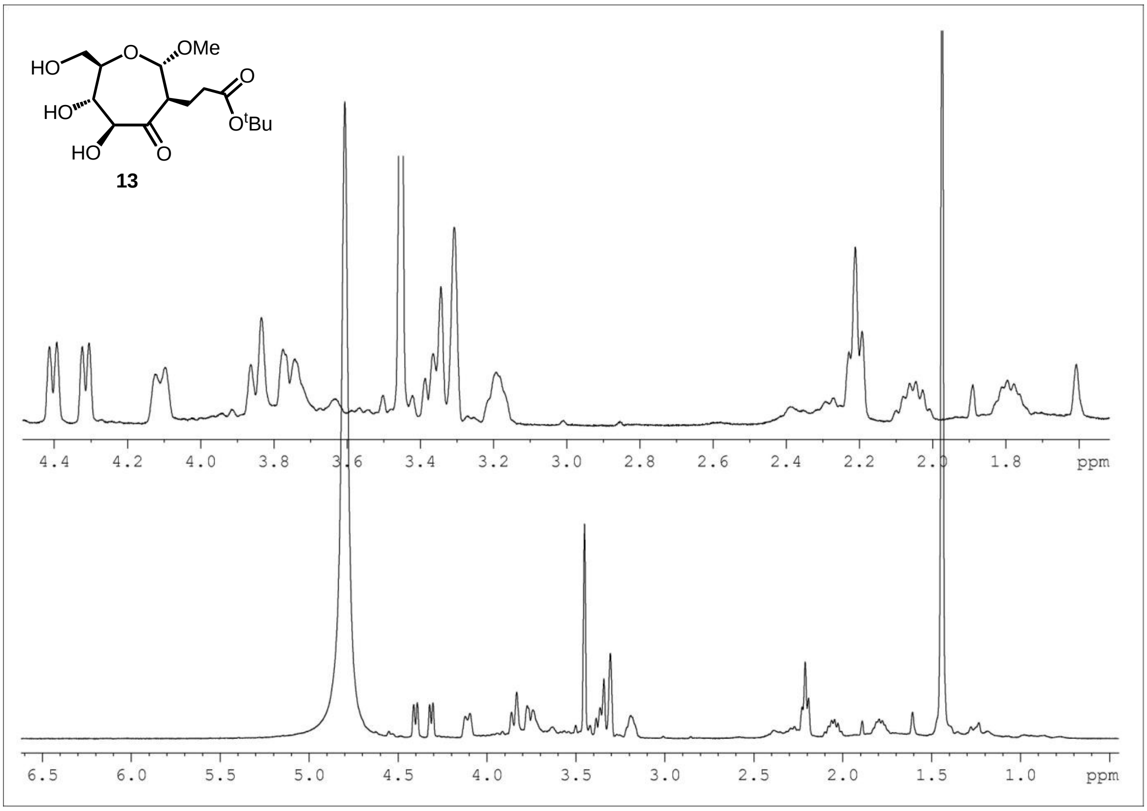

Figure 21: ^{1}H NMR spectrum of **13** (400 MHz, CD_3OD).

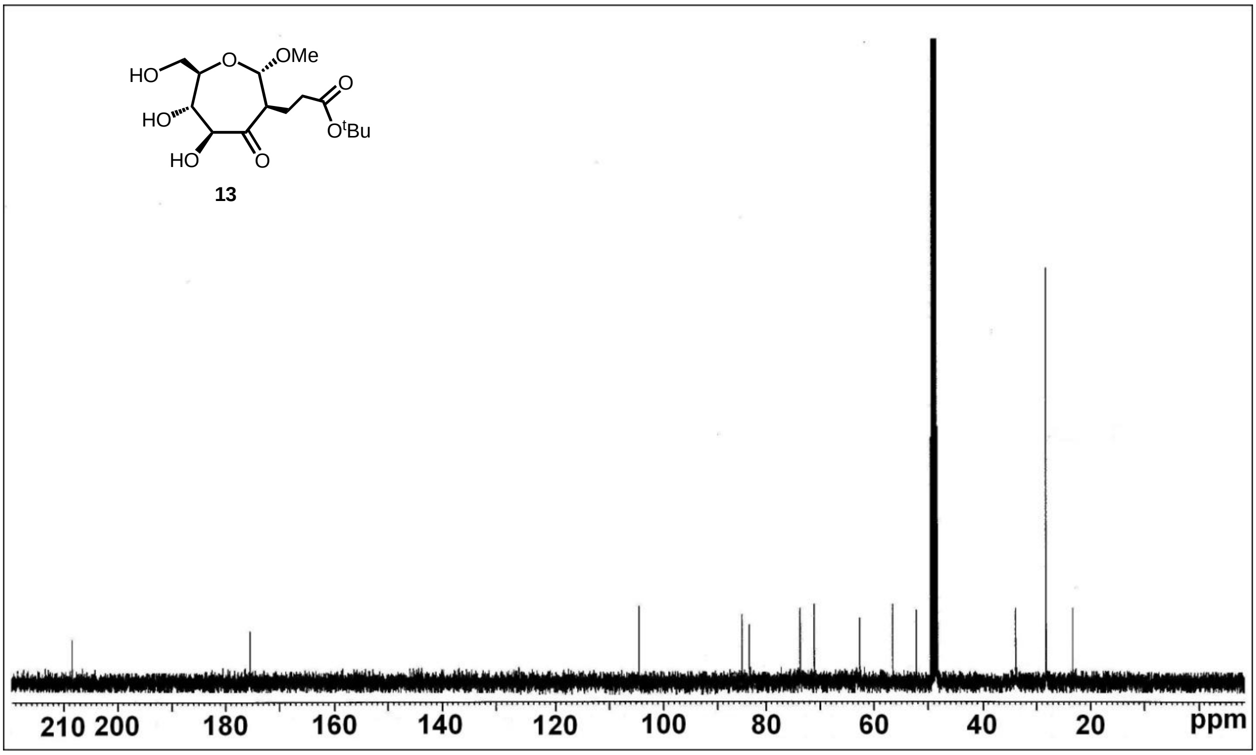

Figure 22: ^{13}C NMR spectrum of **13** (100 MHz, CD_3OD).

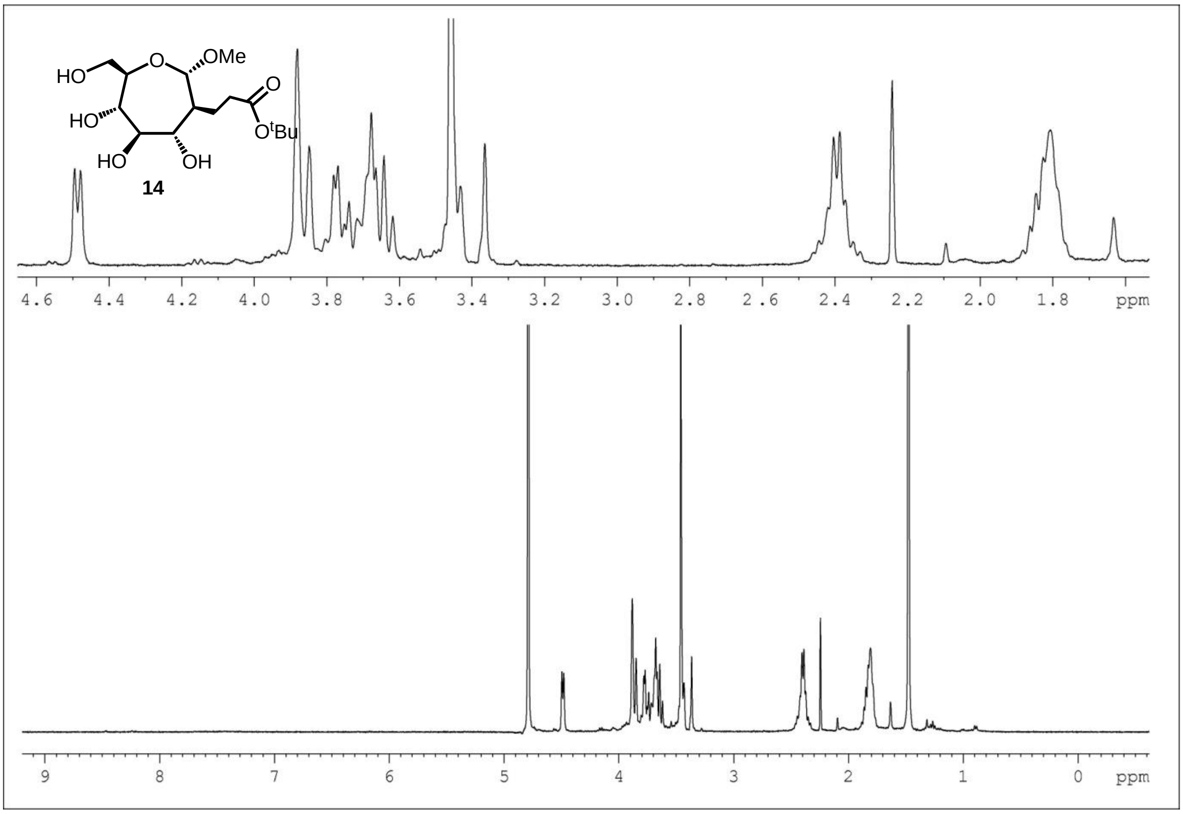

Figure 23: ^{1}H NMR spectrum of **14** (400 MHz, D_2O).

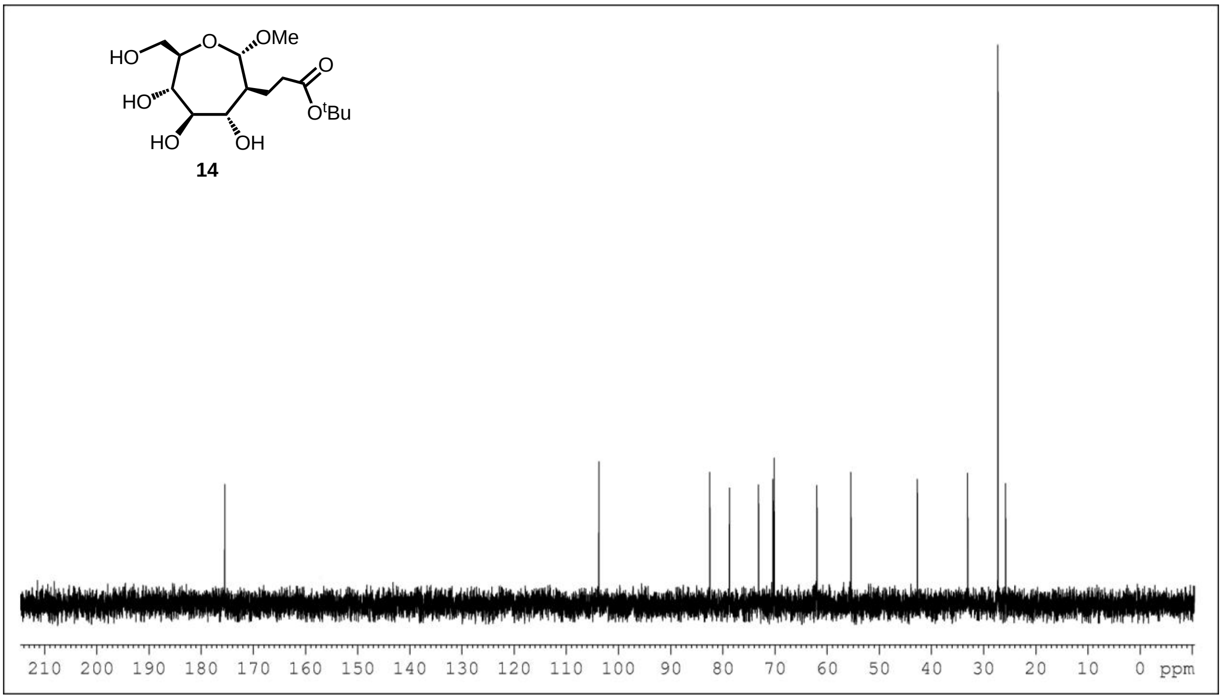

Figure 24: ^{13}C NMR spectrum of **14** (100 MHz, D_2O).

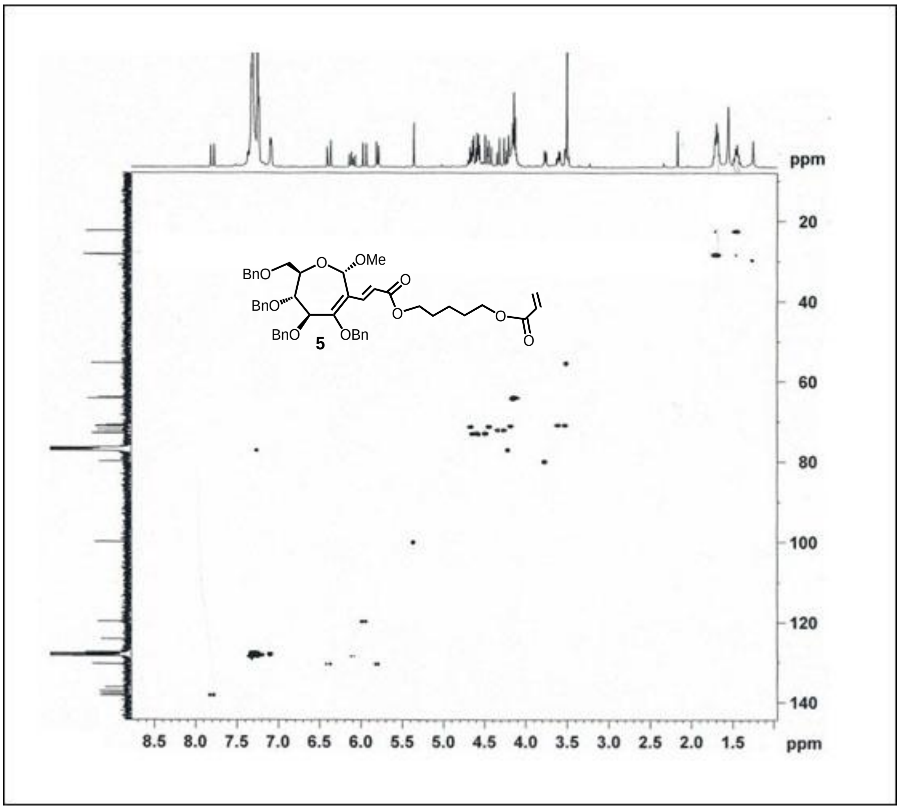

Figure 25: HSQC NMR spectrum of **5** (400 MHz, $CDCl_3$).

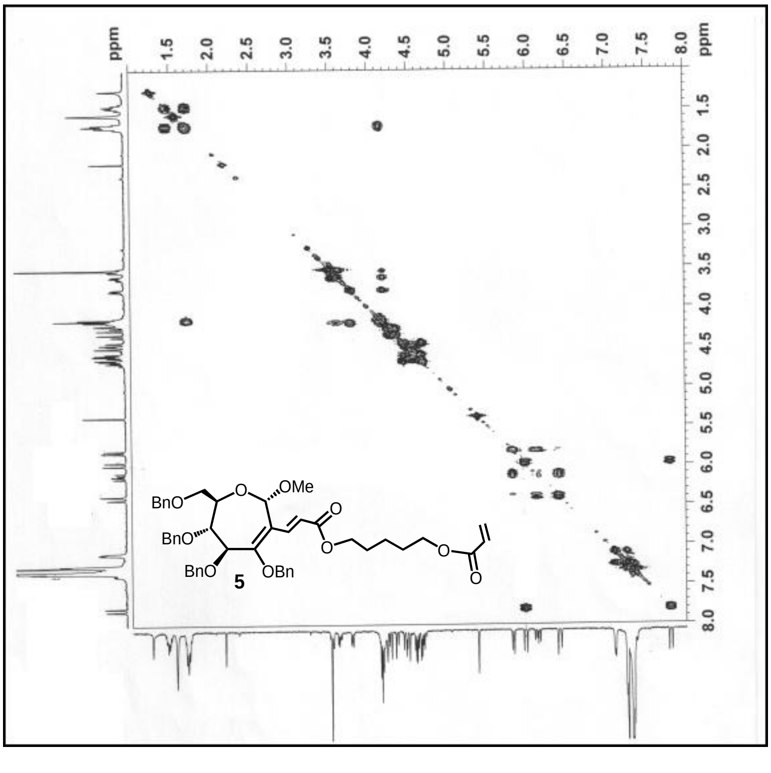

Figure 26: COSY NMR spectrum of **5** (400 MHz, $CDCl_3$).

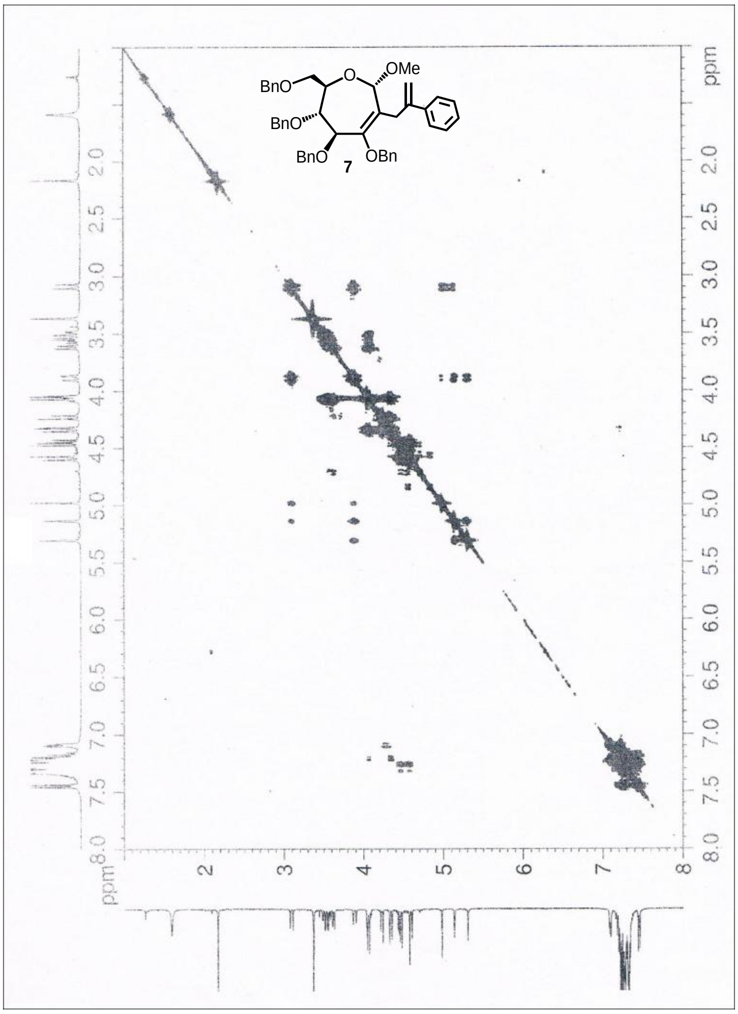

Figure 27: COSY NMR spectrum of **7** (400 MHz, $CDCl_3$).

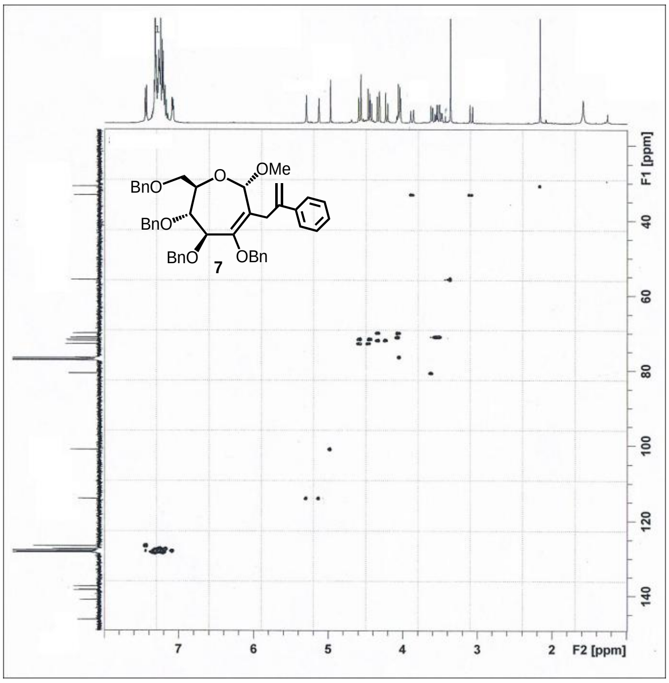

Figure 28: HSQC NMR spectrum of **7** (400 MHz, $CDCl_3$).

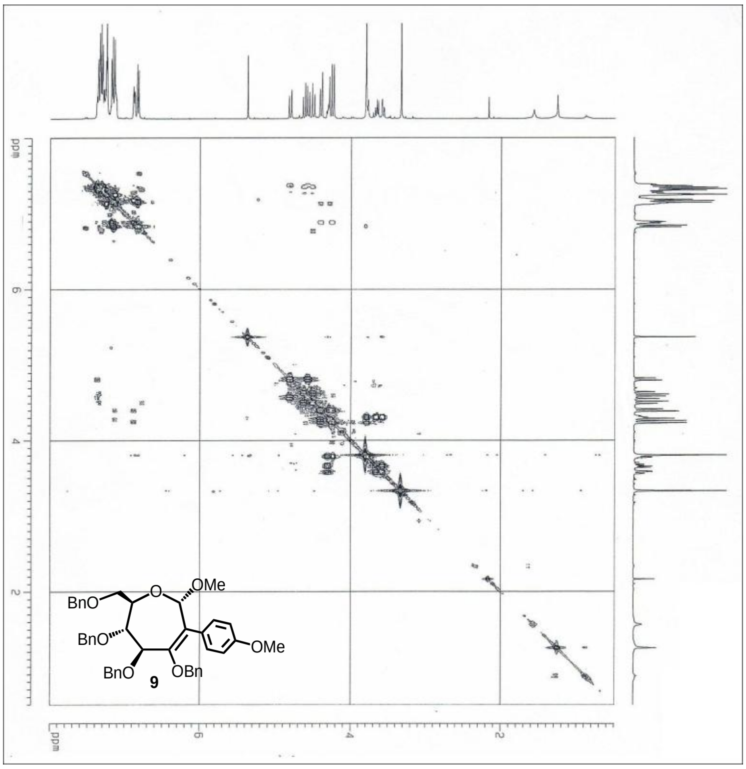

Figure 29: COSY NMR spectrum of **9** (400 MHz, $CDCl_3$).

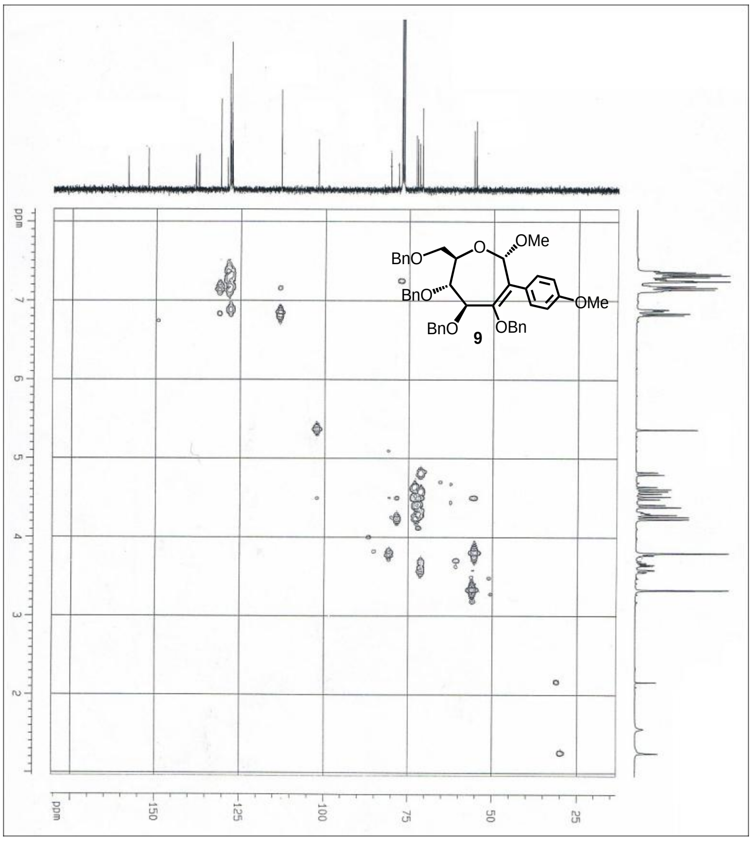

Figure 30: HSQC NMR spectrum of **9** (400 MHz, $CDCl_3$).

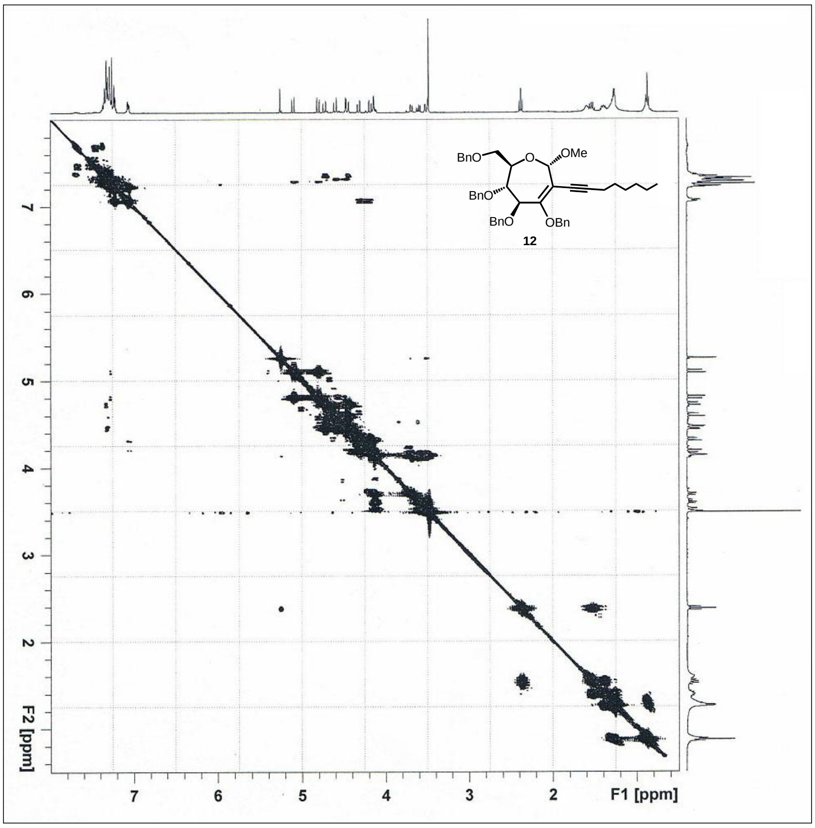

Figure 31: COSY NMR spectrum of **12** (400 MHz, $CDCl_3$).

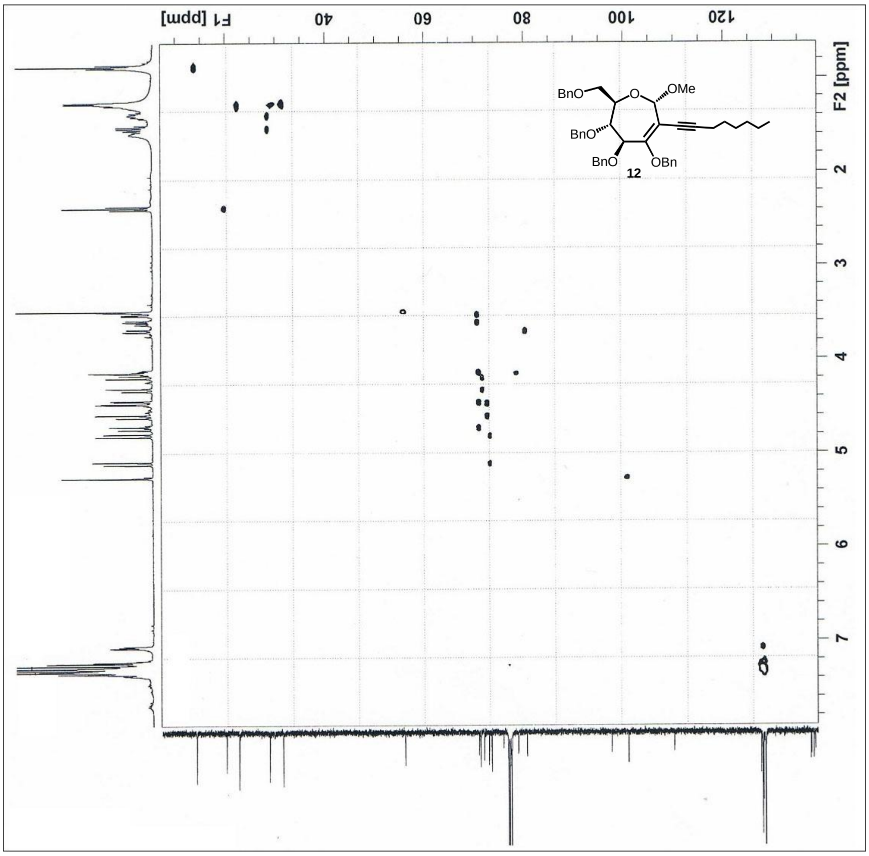

Figure 32: HSQC NMR spectrum of **12** (400 MHz, $CDCl_3$).

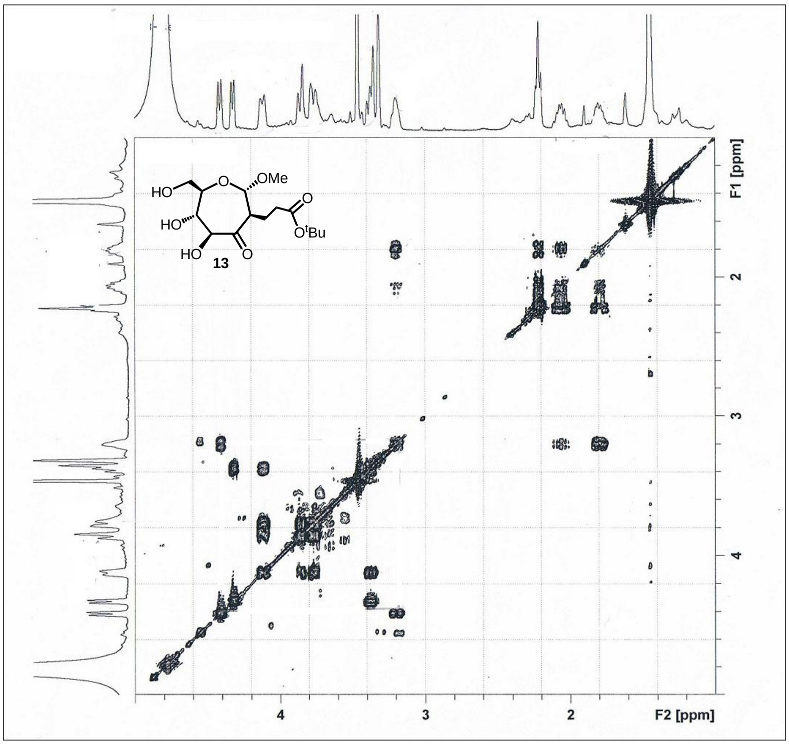

Figure 33: COSY NMR spectrum of **13** (400 MHz, CD_3OD).

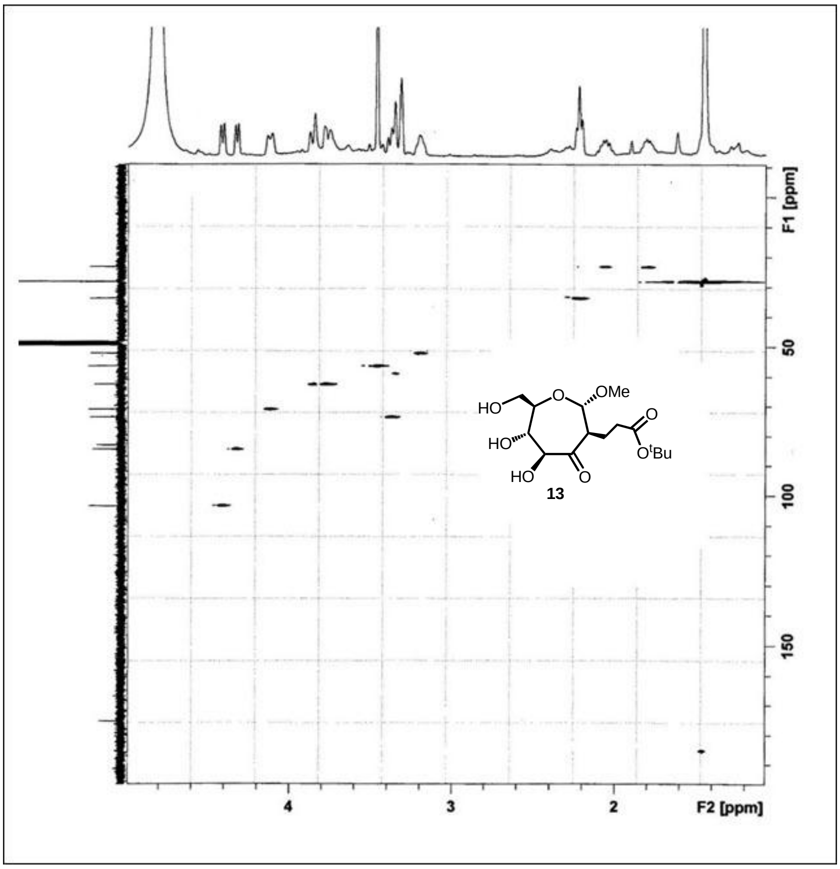

Figure 34: HSQC NMR spectrum of **13** (400 MHz, CD_3OD).

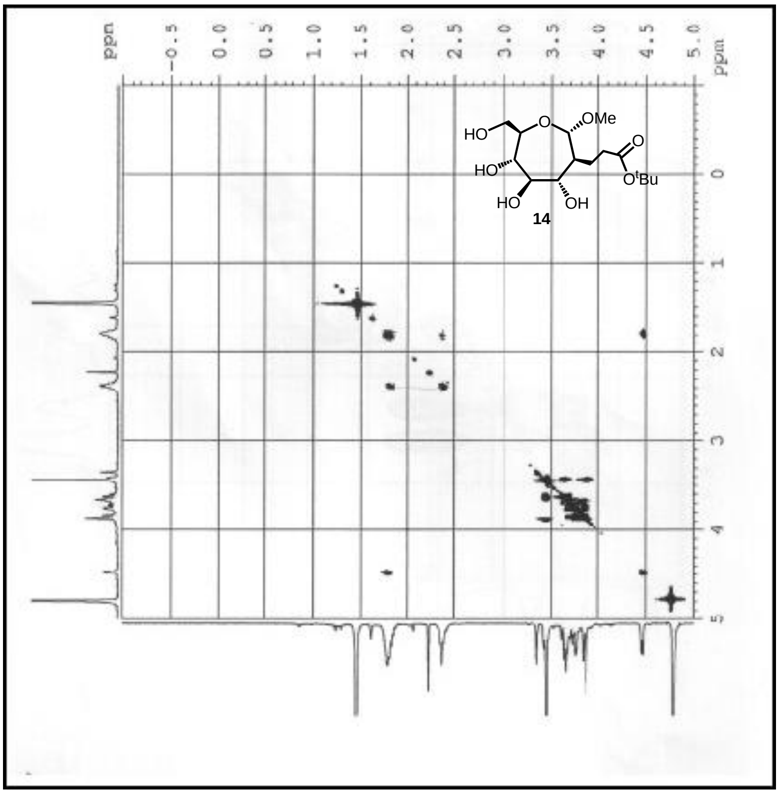

Figure 35: COSY NMR spectrum of **14** (400 MHz, D_2O).

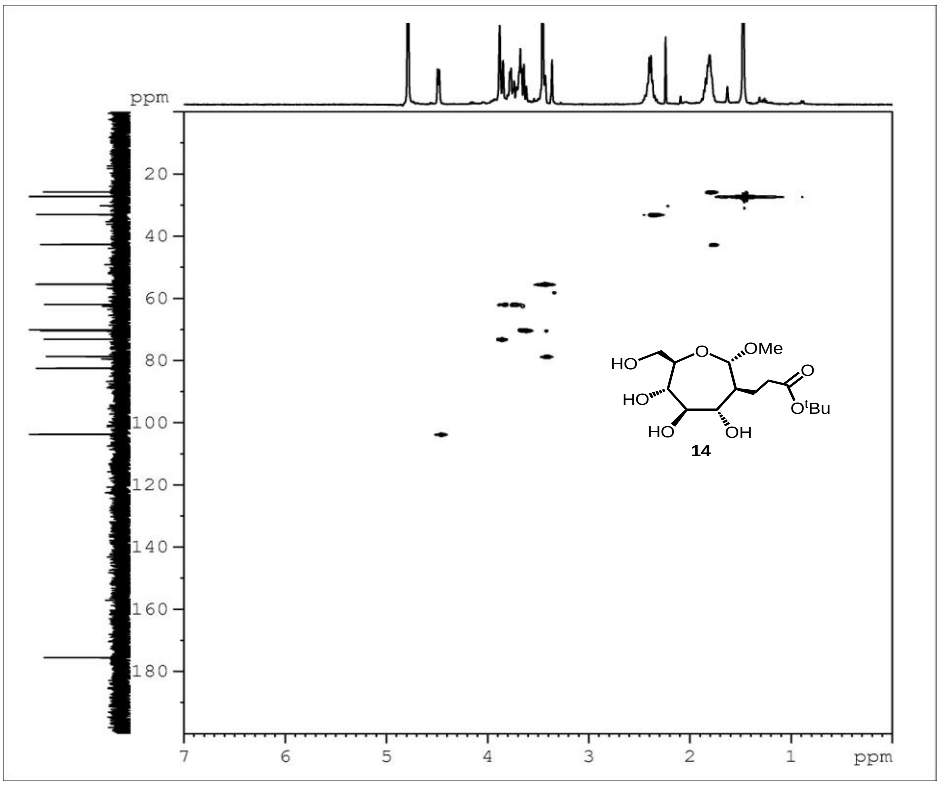

Figure 36: HSQC NMR spectrum of **14** (400 MHz, D_2O).

References

1. Ganesh, N. V.; Jayaraman, N. *J. Org. Chem*. **2007**, *72*, 5500–5504.
